# Identification of Loci Enabling Stable and High-Level Heterologous Gene Expression

**DOI:** 10.3389/fbioe.2021.734902

**Published:** 2021-10-01

**Authors:** Gilles Defrel, Nathalie Marsaud, Etienne Rifa, Frédéric Martins, Fayza Daboussi

**Affiliations:** ^1^ Toulouse Biotechnology Institute (TBI), Université de Toulouse, CNRS, INRAE, INSA, Toulouse, France; ^2^ Toulouse Biotechnology Institute (TBI), Plateforme Genome et Transcriptome (GeT-Biopuces) Université de Toulouse, CNRS, INRAE, INSA, Toulouse, France; ^3^ Institut des Maladies Métaboliques et Cardiovasculaires (I2MC), UMR1297, INSERM, UPS, Toulouse, France; ^4^ Plateforme Genome et Transcriptome (GeT), Genopole Toulouse, Toulouse, France; ^5^ Toulouse White Biotechnology (TWB), INSA, Toulouse, France

**Keywords:** genome engineering, safe harbor, transgene expression, microalgae, biotechnology

## Abstract

Efficient and reliable genome engineering technologies have yet to be developed for diatoms. The delivery of DNA in diatoms results in the random integration of multiple copies, quite often leading to heterogeneous gene activity, as well as host instability. Transgenic diatoms are generally selected on the basis of transgene expression or high enzyme activity, without consideration of the copy number or the integration locus. Here, we propose an integrated pipeline for the diatom, *Phaeodactylum tricornutum*, that accurately quantifies transgene activity using a β-glucuronidase assay and the number of transgene copies integrated into the genome through Droplet Digital PCR (ddPCR). An exhaustive and systematic analysis performed on 93 strains indicated that 42% of them exhibited high β-glucuronidase activity. Though most were attributed to high transgene copy numbers, we succeeded in isolating single-copy clones, as well as sequencing the integration loci. In addition to demonstrating the impact of the genomic integration site on gene activity, this study identifies integration sites for stable transgene expression in *Phaeodactylum tricornutum*.

## 1 Introduction

Diatoms are promising cellular factories for many industrial applications. They serve as a rich source of natural compounds, such as lipids, carbohydrates, and carotenoids, which make them attractive for the nutraceutical, food, feed, and energy markets (Butler et al., 2020). The recent development of genome engineering in certain species has opened up new avenues for the production of drugs, chemicals, biofuels, and materials by introducing heterologous genes or silencing specific ones, enabling redirection of the metabolic fluxes toward the metabolite of interest. Recent studies notably reported on engineering approaches to increase or modify the pattern of carotenoid or lipid content (Trentacoste et al., 2013; Daboussi et al., 2014; Kadono et al., 2015; Eilers et al., 2016) or to produce bioplastics (Hempel et al., 2011a). Other examples highlighted the potential to create algae-based cell factories for therapeutic applications by the delivery of an expression cassette for two antibody chains or the integration of pathway modules in terpenoid biosynthesis (Hempel et al., 2011b; D’Adamo et al., 2019; Fabris et al., 2020). More recently, the development of genome editing technologies has enabled inactivation of one (Daboussi et al., 2014; Nymark et al., 2016; Kroth et al., 2018; Sharma et al., 2018; Stukenberg et al., 2018; Slattery, 2020) or several genes simultaneously (Serif et al., 2018), leading to the generation of strains with improved metabolism (Daboussi et al., 2014; Huang and Daboussi, 2017; Kroth et al., 2018).

In contrast to yeast and bacteria, the use of diatoms as industrial cell factories is beset with the issue of expressing transgenes in a stable and predictable manner. Several parameters can affect protein production, including the level of transgene expression and the potential cellular toxicity and metabolic burden induced by expression of the recombinant protein. It has been shown that the level of transgene expression varies among transformants in mammalian and plant cells according to the transgene copy number and sites of integration (Butaye et al., 2005; Daboussi et al., 2012; Laboulaye et al., 2018). Although chromatin compaction, methylation, and the chromosomal context have been shown to be key factors that influence transgene expression in mammals, plants, and insects (Fagard and Vaucheret, 2000; Daboussi et al., 2012; Gaidukov et al., 2018; Alhaji et al., 2019), little is known about it in microalgae.

In diatoms, transgene integration is classically performed by biolistic transformation and electroporation, which have been successfully used to generate hundreds of transgenic strains (Apt et al., 1996; Miyahara et al., 2013; Huang and Daboussi, 2017). However, these techniques have certain limitations. First, the integration of DNA into the genome occurs randomly and in multiple copies, which can lead to the inactivation of endogenous genes, altered expression of genes near the integration site, or transgene silencing. Second, the transgene copy number is not generally classically measured, as the use of Southern blot analysis, a laborious and time-consuming method requiring relatively large amounts of DNA has been reported in only a few studies, showing variation from 1 to 10 copies (Falciatore et al., 1999; Zaslavskaia et al., 2001; Kira et al., 2016).

Two strategies have been developed to avoid these issues. The first aims to avoid DNA integration into chromosomes by using an episomal vector. This strategy offers the possibility to deliver a low number of plasmid copies and to transiently maintain the plasmids as long as the selection pressure is maintained (Karas et al., 2015; George et al., 2020; Moosburner et al., 2020). Recently, George et al. demonstrated that episomal plasmids lead to homogeneous expression in transformants. By contrast, transformants from random integration were associated with high variability and overall higher expression (George et al., 2020). However, the common view that episomal plasmids are too unstable for large-scale cultivation has precluded the use of *Phaeodactylum tricornutum* as a biological host for industrial applications. The second aim, presented in this study, consists of identifying chromosomal integration sites that enable efficient and stable transgene expression to use them as platforms for heterologous gene expression.

In this study, we describe a screening pipeline, which enables phenotypic characterization of *P. tricornutum* transgenic clones that is both rapid and quantifiable. By coupling this to Droplet Digital PCR and whole genome sequencing, we were able to evaluate the correlation between transgene activity and copy number and, quite importantly, identify several stable loci for transgene insertion.

## 2 Materials and Methods

### 2.1 Culture Conditions

The *P. tricornutum* strain CCMP2561 (NCMA) was grown axenically at 20°C in vented-cap flasks containing silica-free F/2 medium (Sigma G0154) with 40% sea salts (Sigma S9883). Sea salt is an artificial salt mixture closely resembling the composition of the dissolved salts of ocean water (Chloride 19–20 g/L, Sodium 10.7–11 g/L, Sulfate 2.66 g/L, Magnesium 1.32 g/L, Potassium 300–400 mg/L, Calcium 400 mg/L, Carbonate 140–200 mg/L, Boron 5.6 mg/L, Strontium 8.8 mg/L). Incubators (Sanyo, Panasonic model MLR-351) were equipped with white neon light tubes providing illumination of approximately 120 μmol photons m^−2^ s^−1^ and a photoperiod of 12 h light/12 h dark.

### 2.2 Cloning and Genetic Construct Assembly

Native genetic parts were amplified from plasmids using Q5 High-Fidelity DNA Polymerase (New England Biolabs). Where necessary, native genetic parts were made compatible, (i.e., *BsaI* and *BpiI* sites were removed) using specific primers. Golden Gate assembly reactions were performed with restriction enzymes *BsaI* (Thermo Fisher Scientific) or *BpiI* (Thermo Fisher Scientific), and T4 DNAligase (Thermo Fisher Scientific) according to the protocol of the MoClo Toolkit (Addgene kit # 1000000044). Vectors were transformed into chemically competent *Escherichia coli* XL-1 blue (Agilent) as per the manufacturer’s instructions. Transformed cultures were grown at 37°C on LB medium with appropriate antibiotic selection for levels 0 and 1 vectors from the MoClo Toolkit (Addgene kit # 1000000044) which are respectively destination vectors for single genetic element and assembled transcription unit as outlined in (Weber et al., 2011). Four final vectors were constructed where *uidA* and *NAT* genes, separated by a 2A peptide, are under control of the *Phaeodactylum* pFcpB promoter/pFcpA terminator.

### 2.3 Biolistic Transformation of Polycistronic Vectors

*Phaeodactylum tricornutum* cells (1.5  ×  10^8^ total) were collected from exponentially growing cultures and spread onto 1% agar plates containing F/2 medium with 20 g L^−1^ sea salt (Sigma S9883). Transformations were carried out 24 h later using the microparticle bombardment method adapted from (Apt et al., 1996) with minor modifications as follows. Gold particles (0.6 µm diameter, BioRad) were coated with DNA using 1.25 M CaCl_2_ and 20 mM spermidine. As a negative control, beads were coated with 5 µg NAT selection plasmid and 5 µg empty vector. For each polycistronic cassettes (NAT-T2A-GUS, GUS-T2A-NAT, NAT-P2A-GUS, or GUS-P2A-NAT), beads were coated with 5 µg of DNA. A burst pressure of 1,550  psi and a vacuum of 25 Hg were used.

### 2.4 Selection Procedure for Algal Transformants

For the nourseothricin (NAT) selection procedure, *P. tricornutum* cells transformed with the NAT selection plasmid were collected two- or four-days post-transformation and spread on two F/2 agar plates with 300 μg ml^−1^ NAT (Werner Bioagents). After 3 weeks, colonies were re-streaked on fresh 10-cm 1% agar plates containing F/2 medium with 20 g L^−1^ sea salt (Sigma S9883) and 300 μg ml^−1^ NAT. NAT-resistant clones were picked and transferred into a sterile 96-well plate with fresh medium changed once every 3 weeks. For each assay (GUS assay, ddPCR, MUG assay, and RT-ddPCR), clones were transferred and grown in vented-cap flasks containing silica-free F/2 medium (Sigma G0154) with 40% sea salt (Sigma S9883) at a cell density of 2–4 million cells per ml and in a volume ranging from 10 to 50 ml.

### 2.5 Algal Genomic DNA Extraction

Genomic DNA was extracted from exponentially growing cultures using the NucleoSpin DNA RapidLyse (Macherey-Nagel) protocol. Genomic DNA concentration was measured using a Qubit fluorometer (Thermofisher).

### 2.6 Algal RNA Extraction

Cell cultures were grown in flasks to exponential state. Pellets were collected by centrifugation at 3,000 g for 10 min. Then, pellets were washed with 0.1 M PBS pH 7.4 for 1 min at 20,000 g, followed by flash freezing in liquid nitrogen. Total RNA extraction was performed by classical TRIzol/chloroform isolations and precipitation by isopropanol. RNA concentration was measured using a NanoDrop 2000 spectrophotometer (Ozyme) and the RNA quality was assessed using a Bioanalyzer 2100 (Agilent Technologies). Then, 1 µg total RNA was reverse transcribed using the “High-Capacity cDNA Reverse Transcription” (Thermo Fisher Scientific) protocol. A negative control (without reverse transcriptase enzyme) was prepared for each sample.

### 2.7 Droplet Digital PCR *via* TaqMan

Prior to ddPCR, genomic DNA was digested with a restriction enzyme *HindIII* (New England Biolabs). Digestion was performed using 50 ng of genomic DNA, 1× CutSmart restriction buffer (New England Biolabs), and 10 units *HindIII-HF* restriction enzyme (New England Biolabs) in a total reaction volume of 50 µl for 1 h at 37°C. An aliquot of the restriction reaction was diluted with water to 0.08 ng/μl. The ddPCR reaction mixture was prepared from a QX200™ ddPCR™ Supermix for Probes (No dUTP), 900 nM of primers, and 0.7 ng of DNA template. Ultrapure water was added to a total volume of 22 μl. Two probes were used for each strain: one probe targeting the reference housekeeping gene, *RPS*, and one probe for the *uidA* or *NAT* genes. Therefore, two reactions were ran for each clone: *RPS/uidA* and *RPS/NAT*. The fluorescence signal detected by the ddPCR machine for each target was converted into a concentration in copies/µl. Overall, concentrations of the reference gene were consistent in all clones. However, data processing by modeling according to Poisson’s law makes it possible to get rid of these inter-strain differences as long as the concentrations of the reference genes remain identical within the same strain. Therefore, transgene copy number was determined by normalization with reference gene as follow: Copy number = (targeted gene (copies/µl)/reference gene (copies/µl)) × 2. The probes are listed in Supplementary Figure S16.

### 2.8 RT-ddPCR *via* TaqMan

The ddPCR reaction mixture was prepared from a QX200™ ddPCR™ Supermix for Probes (No dUTP), 900 nM primers, and 25 ng cDNA template (the cDNA quantity was based on the initial amount of RNA used for the reverse-complementation). Ultrapure water was added to a total volume of 22 μl. The following steps were performed as described above.

### 2.9 Histochemical Assay (GUS Screening)

*Phaeodactylum tricornutum* cells (1 × 10^6^) were collected from exponentially growing cultures and centrifuged at 3,000 g for 10 min. Cells were resuspended with 20 µl fresh medium and then spread onto 1% agar in 24-well plates containing F/2 medium with 20 g L^−1^ sea salts (1 ml/well). Cells were incubated for 4 days in incubators. On the day of screening (5th day), fresh GUS buffer was prepared (0.1 M NaP pH 7, 0.5 M KFe_3_ [CN_6_], 0.5 M KFe_4_ [CN_6_], 1 mg/ml X-Gluc). Then, 150 µl fresh GUS Buffer was added to each well and the plates incubated at 37°C for 24 h. An image of the 24-well plates was captured every 2 h for 10 h using a scanner. A final image was captured after 24 h of incubation. Quantification of the β-glucuronidase activity was made by converting the color intensity into a grayscale, from zero (black) to 255 (white), using ImageJ software. The T_0_ and T_6_ photos were uploaded onto the software and converted into 8-bit pictures. An identical scale of 40.4 pixels/mm was defined to obtain homogeneous data. A circular selection covering the interior surface of a well was used to measure 1) the area, 2) minimum grey level, 3) maximum grey level, and 4) mean grey level. The mean grey values were used to determine the delta grey value: Δ grey (T_0_–T_6_). The wild type delta was assigned zero and the Gus5 delta 100 as the reference strains. Transformants with a delta equivalent to that of wild type were assigned a value of zero. Delta values were then converted into a percentage relative to the value of the Gus5 strain.

### 2.10 Measurement of GUS Activity

GUS activity was measured by monitoring the cleavage of 4-MUG (4-methylumbelliferyl-beta-D-glucuronide) to 4-MU (4-methyl umbelliferone) by the β-glucuronidase enzyme. 4-MUG and 4-MU were purchased from Sigma-Aldrich (Oakville, ON, Canada). Two assay buffers, 10 µM (100 µM MUG, 1 M NaP pH 7) and 1 µM (10 µM MUG, 1 M NaP pH 7), were added to 20 ng crude protein extracts in black 96-well plates (Thermo Fischer Scientific; 237108). Ultrapure water was used to obtain a final volume of 200 µl. The formation of 4-MU was measured using an Infinite M200 Pro plate reader (Tecan), with excitation at 370 nm and emission at 450 nm. A measurement was made after the addition of the crude protein extracts and then the plate was incubated at 37°C for 10 h. Measurements were taken after 1, 2, 4, 6, 8, and 10 h of incubation. All results are presented as the mean (±SD) of at least three replicates.

### 2.11 Genomic DNA Extraction and Whole-Genome Sequencing

Extraction and purification of genomic DNA were performed according to the protocol of (George et al., 2020). High molecular weight (HMW) genomic DNA from *P. tricornutum* transformants was extracted using 2 × 10^8^ cells to obtain ∼1.5 μg HMW purified gDNA (dx.doi.org/10.17504/protocols.io.qzudx6w). After DNA precipitation, an additional 1X purification step was performed via the AMPure XP beads (Beckman). The DNA was resuspended in 30 μl Elution Buffer. Quality control of the samples was performed by NanoDrop 2000 spectrophotometer (Ozyme) and Qubit Fluorometer DNA HS assay (Thermo Fisher). After extraction, 1 µg of HMW DNA was fragmented 1 min at 7,200 rpm in Eppendorf 5424 with Covaris g-Tube (protocol Shearing genomic DNA using the Covaris g-TUBE™ September 03, 2019 from Oxford Nanopore Technologies). The size of fragmented DNA was checked by Bioanalyzer with the DNA12000 kit (Agilent Technologies). DNA fragments were between 8 and 10 kb. Whole genome sequencing was carried out by MinION (Oxford Nanopore Technologies).

MinION sequencing libraries were prepared according to the 1D Genomic DNA by Ligation (SQK-LSK109) protocol supplied by the MinION manufacturer (Oxford Nanopore Technologies). Five samples were barcoded and sequencing 24 h on the FlowCell.

The raw data in fast5 format were generated by the MinKNOW software (version 3.6.5). Then, demultiplexing and base calling were performed using Guppy software (version 3.6.0) to obtain a sequence file in fastq format for each sample. A quality control of the data was then performed using Nanoplot and fastqc. Then, flye (version 2.8.3) (Kolmogorov et al., 2019) and wtdbg2 (version 2.5) (Ruan and Li, 2020) were used for the *de novo* assembly of the sequences. The quality of the assemblies was evaluated with Quast (Version: 5.0.2) (Gurevich et al., 2013). The plasmids used for transformation were sought in the various assemblies by alignment with blast (version 2.10.1) to determine the insertion position. Finally, the flanking regions of the integrated plasmid were aligned to the NCBI reference (NC_011678.1) using blast (https://www.ncbi.nlm.nih.gov/pmc/articles/PMC441573/) for identification of the insertion loci in the *P. tricornutum* reference strain.

### 2.12 Statistical Analysis

For transgene copy number, Wilcoxon test was used to compare copy number determined by ddPCR (R software). For RT-ddPCR (Figure 6), *t*-test was used to compare the means of the different conditions. A difference was statistically significant when *p*-value < 0.05. Error bars in figures represent standard deviation (SD) of the means of two independent experiments (Figure 5; Supplementary Figure S5) or three independent experiments (Figure 6; Supplementary Figure S10).

## 3 Results

### 3.1 Designing a Polycistronic Expression System

**FIGURE 1:**
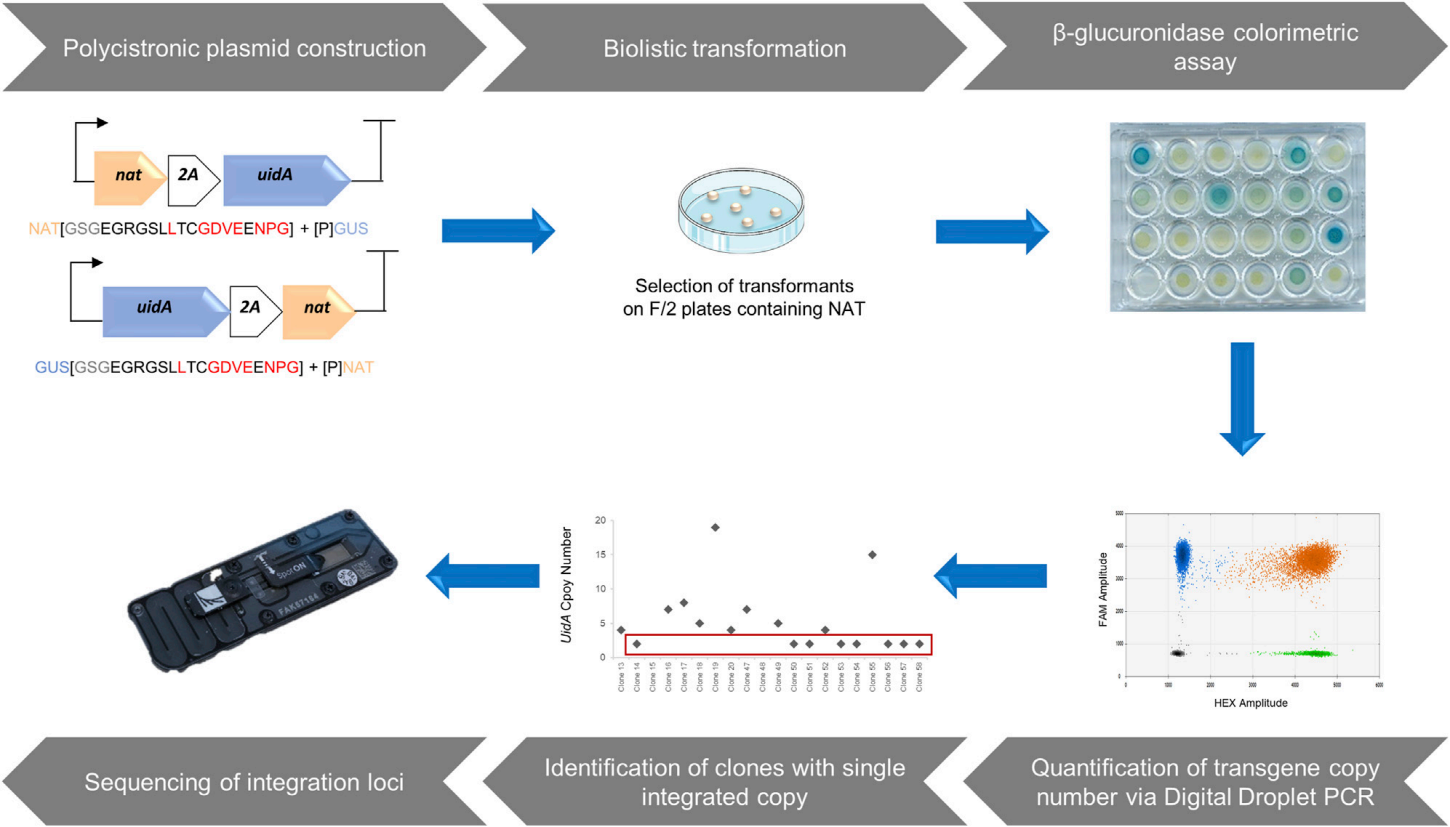
A Step-by-step approach to identify loci enabling a high-level of heterologous gene expression. The design of polycistronic expression vectors using modular cloning system adapted from Weber et al. (2011). Polycistronic constructions were delivered into the *P. tricornutum* genome through biolistic transformation and the selection of transformants was based on antibiotic resistance. Phenotypic characterization of clones from the transformation was performed *via* β-glucuronidase colorimetric assay based on Falciatore et al. (1999). Assessment of the copy number of integrated transgenes was measured by a rapid, sensitive, and robust method, droplet digital PCR, allowing the identification of clones harboring a single copy of the transgene. After molecular characterization of single copy clones, those with a complete polycistronic cassette were sequenced to identify chromosomal integration sites that allow a high level of transgene expression.

The first step towards the development of the pipeline was to design the ideal transgene expression cassette (Figure 1). To ensure transformation with a single vector, we opted for a polycistronic expression system that allows the simultaneous expression of multiple genes by generating a single mRNA to be processed for the delivery of several proteins. Polycistronic genes are uncommon in eukaryotes but are made possible through the use of IRES (Internal Ribosome Entry Sites) and type 2A cleavage peptides, both used in molecular biology (Szymczak and Vignali, 2005). IRES are large sequences (>600 nucleotides) that do not necessarily ensure equivalent expression of the various genes because of their increasing distance from the transcription initiation site. In contrast, 2A peptides identified in viral genomes, including P2A derived from *porcine teschovirus-1* 2A and T2A derived from *thosea asigna virus* 2A, are short sequences (18–22 amino acids) (de Felipe et al., 2003) that allow the cleavage of polypeptides during eukaryotic translation via ribosomes due to the presence and recognition of a highly conserved motif, GDVEXNPGP. The cleavage pattern is illustrated in Figure 1. Due to their short size, good cleavage rate, and relatively high levels of downstream protein expression, many researchers have rapidly adopted 2A self-cleavage peptides as tools to provide high amounts of the co-expressed proteins (Szymczak et al., 2004; Yasuda et al., 2005; Trichas et al., 2008; Kim et al., 2011; Slattery et al., 2018).

Here, we opted for the creation of a polycistronic cassette composed of two genes, generating an easily observable and quantifiable phenotype. The first gene corresponded to the *NAT* gene of *Streptomyces noursei*, conferring resistance to nourseothricin (NAT), which has proven to be an effective selection marker in *P. tricornutum* (Falciatore et al., 2020). Moreover, unlike bleomycin or phleomycin antibiotics, it does not generate double-stranded breaks in the genome. The second gene, *uidA* from *E. coli*, encoding β-glucuronidase, was chosen as a reporter gene because the resulting phenotype generates a blue color, of varying intensity depending on the reporter activity. The resulting phenotype is easily observable and measurable, and has been shown to be usable in *P. tricornutum* (Falciatore et al., 1999; Zaslavskaia et al., 2001; De Riso et al., 2009). This polycistronic cassette enhances the probability that antibiotic-resistant clones will also produce the protein of interest. It should allow the selection of clones with a single integrated copy, which is crucial for the identification of loci that enable high and stable transgene expression. In this simplified polycistronic cassette, the *uidA* and *NAT* genes were separated by T2A or P2A, 2A peptides known for their efficiency. To avoid a possible negative position effect, two constructs were developed for each 2A peptide, in which the position of the two genes was alternated, resulting in four constructs: GUS-T2A-NAT (GTN), GUS-P2A-NAT (GPN), NAT-T2A-GUS (NTG), and NAT-P2A-GUS (NPG).

### 3.2 β-Glucuronidase Activity in *Phaeodactylum tricornutum* Transgenic Strains

**FIGURE 2:**
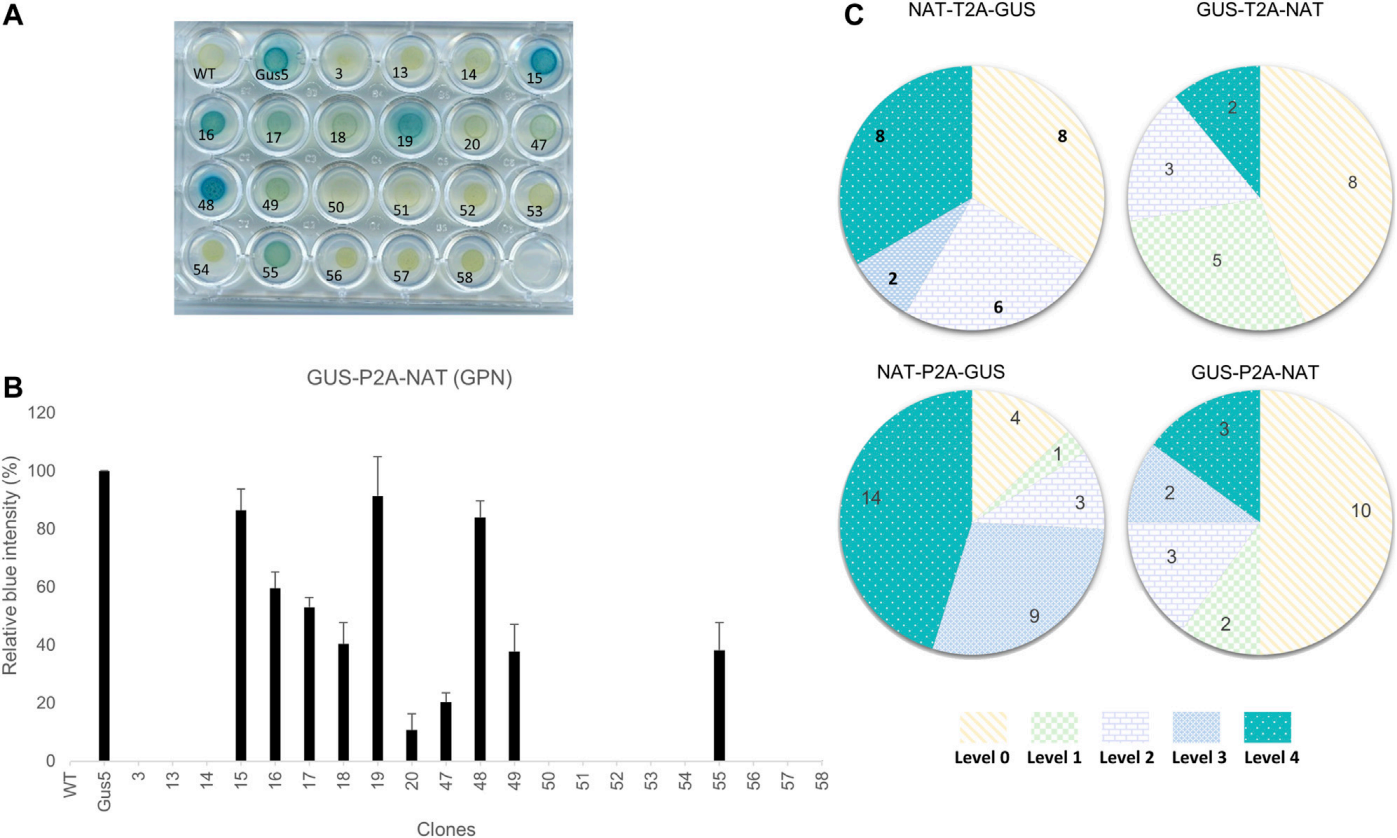
Phenotypic characterization of transformants and classification based on color intensity. **(A)** Example of the β-glucuronidase activity assay on the 20 clones from the GUS-P2A-NAT construct. Gus5, as the reference strain, was given a value of 100% and wild type, as well as strains from transformation with NAT vector (clone 3), were given a value of 0%. **(B)** Bar chart of the relative color intensity calculated for the GUS-P2A-NAT clones. The presented values are averages of the relative color intensity at 24 h for three independent colorimetric experiments. **(C)** Distribution of GUS intensity in the groups. Clones were assigned to a level based on their relative color intensity as follows: Level 0 (0%), Level 1 (1–25%), Level 2 (25–50%), Level 3 (50–75%), and Level 4 (>75%). Values presented in the diagrams are the number of clones assigned to each level for each group.

*Phaeodactylum tricornutum* cells were transformed independently with four different constructs using biolistics. After 3-weeks, several dozen clones were picked and four groups defined, each consisting of 25% total NAT-resistant clones obtained after transformation, and subjected to the characterization pipeline described above. The first step of characterization consisted of evaluating expression of the transgene in clones based on β-glucuronidase activity. We adapted a protocol established by Falciatore et al. (1999). Cells were spread onto 24-well agar plates for 4 days and incubated with the chromogenic substrate X-Gluc (5-bromo-4-chloro-3-indolyl-β-D-glucuronide). The hydrolysis of X-Gluc by β-glucuronidase produces glucuronic acid and indoxyl 5-bromo-4-chlorine, which is transformed into a deep blue indigo dye by oxidation. Twenty-four hours of kinetic monitoring showed the appearance of blue clones, allowing discrimination between clones with detectable β-glucuronidase activity, called GUS (+), from clones without activity, called GUS (−). We also observed varying color intensity within the groups from a light blue-green to a deep blue phenotype (Figure 2A). We quantified the color intensity by converting color images to grayscale. The Gus5 strain was used as a reference to normalize the grayscale for all experiments. Color intensity is expressed as the percentage from 0%, similar to wild type, to over 100%. Five classes of color intensity were defined based on β-glucuronidase intensity: level 0 (0% color intensity) regrouping the GUS (−) clones, level 1 (<25%), level 2 (25–50%), level 3 (50–75%), and level 4 (>75%) (Figures 2B,C). An example of the distribution of color intensity is shown in Figure 2B. The distribution for the other constructs is presented in Supplementary Figure S5.

Overall, 93 NAT-resistant colonies transformed with polycistronic vectors were analyzed. All colored plates arising from β-glucoronidase activity of transgenic clones are presented in Supplementary Figures S1–S4. From 50 to 87% of the NAT-resistant colonies showed detectable activity, depending on the condition. GUS (−) clones represented 44 and 50% of the total when the *uidA* gene was located in the first position in the polycistron cassette (GTN and GPN, respectively) and 13 and 33% when *uidA* was located in the second position (NPG and NTG, respectively) (Figure 2C).

We observed different patterns of β-glucuronidase intensity among GUS (+) clones, depending on the gene order in the construct (Figure 2C; Supplementary Figure S5). When the *uidA* gene was in the second position, level 3 and 4 clones were highly represented, with 10 of 24 for NTG and 23 of 31 for the NPG group. On the contrary, this proportion was markedly lower when the *uidA* gene was in first position in the polycistronic cassette. Indeed, in the GTN condition, only 2 of 18 clones were classified as level 4 and for GPN, only 5 of 20 clones corresponded to level 3 or 4 (Figure 2C). One possibility is that the 2A peptide sequence C-terminal to the β-glucuronidase protein affects its activity. Indeed, the upstream protein of 2A is left with a polypeptide of approximately 20 amino acids at its C-terminus after cleavage, while the downstream protein has an additional proline at its N-terminus.

In addition to observed differences in color intensity, we also observed differences in the time of appearance of the color. Most high-activity clones (level 3 and 4) exhibited blue coloration within the first 6 h, regardless of the group. For example, such clones represented 15 of 23 clones for NPG and 3 of 5 clones for both GTN and GPN, whereas they represented slightly less than half of the level 3 and 4 population in NTG, with 6 of 10 clones (Supplementary Figure S6).

Overall, these results demonstrate that polycistronic constructions can drive multiple gene expression, here NAT resistance and β-glucuronidase gene. At least 50% of the NAT-resistant clones showed a detectable blue phenotype GUS (+). Moreover, our results also suggest a position effect, affecting the GUS (+)/GUS (−) ratio and the distribution of color intensity.

### 3.3 Determination of Transgene Copy Number by Droplet Digital PCR

**FIGURE 3:**
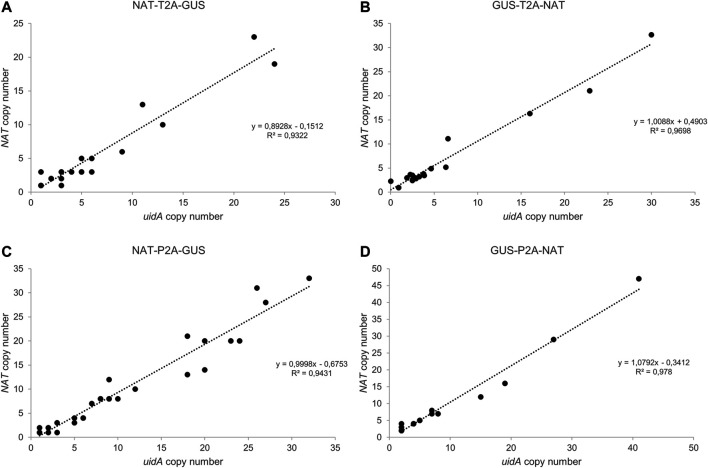
Droplet Digital PCR (ddPCR): a robust technology to determine transgene copy number. **(A–D)** Correlation between *uidA* and *NAT* copy number in groups **(A)** NAT-T2A-GUS, **(B)** GUS-T2A-NAT, **(C)** NAT-P2A-GUS, and **(D)** GUS-P2A-NAT. The copy number for *uidA* and *NAT* was determined for each clone using Droplet Digital PCR (ddPCR). Data were analyzed using QuantaSoft Bio-Rad software.

**FIGURE 4:**
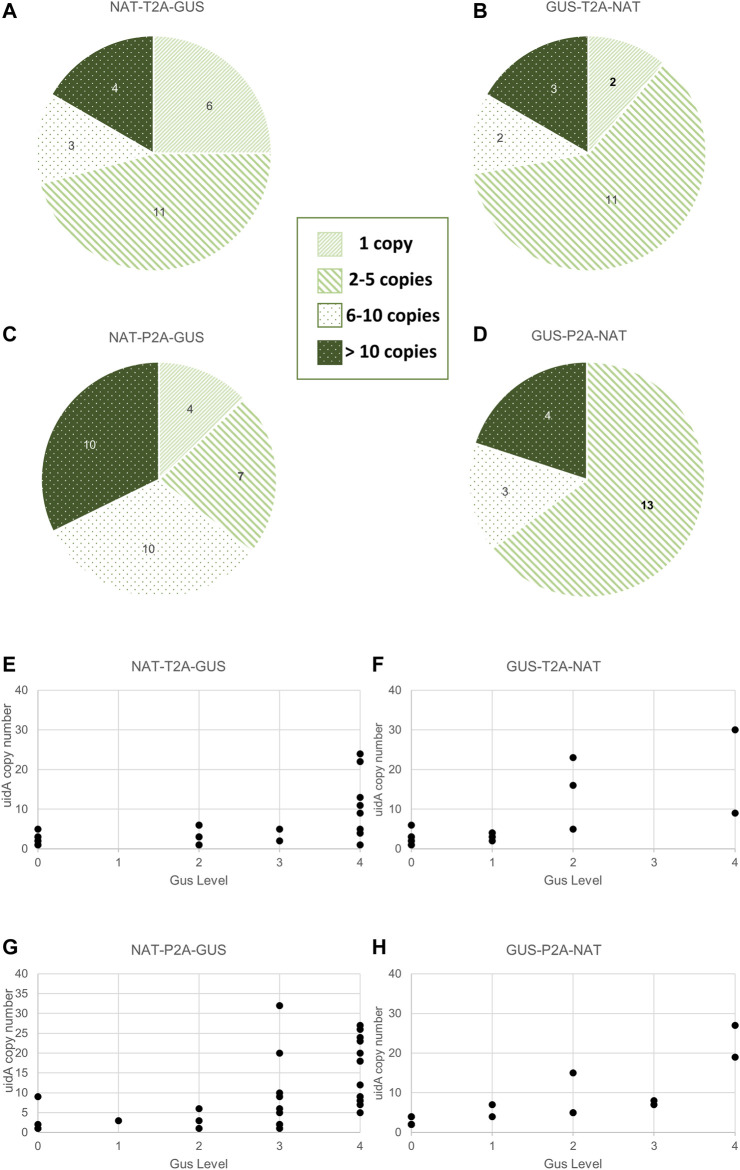
Relationship between transgene copy number and GUS intensity in the various groups. **(A–D)** Distribution of *uidA* copy number in the various groups. Clones were classified according to the following five categories: 0 copy, 1 copy, 2 to 5 copies, 6 to 10 copies, and over 10 copies. Values indicated in the pie chart are the number of clones assigned to the category. A statistical analysis was performed on the groups and revealed no difference between all groups except for NTG and NPG (Wilcoxon test, *p*-value = 0.02). **(E–H)** Distribution of *uidA* copy number according to the color intensity in the various groups. The *uidA* copy number (*y*-axis) determined via ddPCR and the GUS intensity level associated (*x*-axis) for each clone of the four working groups are shown.

An important goal of our project was to determine whether there is a correlation between *uidA* copy number and β-glucuronidase intensity. Thus, we developed a high-throughput screening method to quantify the transgene copy number based on Droplet Digital PCR (Figure 3; Supplementary Figure S7). This technology has been applied in plant engineering to accurately measure transgene copy number in crop species with a wide range of genome sizes (Głowacka et al., 2016; Xu et al., 2016; Collier et al., 2017; Sun and Joyce, 2017). We designed several pairs of primers and a probe to detect a unique sequence in the delivered transgene, one targeting the *NAT* gene and another targeting the *uidA* gene. Primers and probes to detect two endogenous reference genes (*TBP* and *RPS*, encoding the TATA-binding protein and the 30S ribosomal protein subunit, respectively) were designed and tested for their ability to detect endogenous sequences (Supplementary Figure S17). Both targeted gene amplicons were detected using a FAM™-labeled probe, while reference gene amplicons were detected with a HEX™-labeled probe. The number of positive droplets is used to calculate the fraction of positive partitions, from which a concentration (copies/µl) is estimated by modeling as a Poisson distribution with 95% confidence intervals. In the same ddPCR reaction, we mixed the primers and probes designed to detect the reference *RPS* and the *uidA* transgene to evaluate the copy number in the transgenic strains (Figure 3). We performed the experiment on all transgenic strains. The copy number varied from 1 to 41 (Figures 3, 4). Overall, these results demonstrate that duplexed ddPCR is a powerful tool to detect one, two, or more transgene copies.

### 3.4 Analysis of the Relationship Between *uidA* Copy Number and β-Glucuronidase Activity

Transgene copy number measurements were measured on all (93 strains) transgenic strains derived from the groups described in Figure 2C and Supplementary Figure S8. Furthermore, most clones had a *uidA* copy number ≤5 copies within the NTG (71%), GTN (72%), and GPN (65%) groups. This proportion dropped to 35% for the NPG group, for which approximately 65% of the population had ≥6 copies of *uidA* integrated (Figures 4A–D). A Wilcoxon test was performed on *uidA* copy number in the different groups and revealed a significant difference between NTG vs. NPG, *p*-value = 0.02). These results confirm that most of the transformed clones integrated no more than 6 transgene copies, as formerly demonstrated in the literature (Falciatore et al., 1999; Zaslavskaia et al., 2001; Zhang and Hu, 2014; D’Adamo et al., 2019). Interestingly, certain clones showed more than 20 integrated copies within their genome (up to 41 copies) (Supplementary Figure S8). Such high transgene copy numbers have not been reported for *Phaeodactylum tricornutum*.

We wished to determine whether there is a potential correlation between copy number and β-glucuronidase activity. Thus, we first analyzed the *uidA* copy number in transgenic strains with high and rapid β-glucuronidase activity. All of the NPG, GTN, and GPN clones and 83% (5 out of 6) of the NTG clones showing a high level of β-glucuronidase activity (level 3 and 4) within the first 6 h appeared to have 6 integrated copies or more (Supplementary Figure S6). However, the opposite was not true, as the distribution of *uidA* copy number according to color intensity showed level 3 and 4 clones with <6 transgene copies (clones 69 (NTG), 78 and 84 (NPG), clones 95 (NPG), 30 and 72 (NTG), as well as level 0, 1, and 2, clones with ≥6 copies (clones 44 (GTN), clone 47 (GPN), clones 34 (NTG) and 39 (GTN), (Figures 4E–H; Supplementary Figures S12–S15). Furthermore, several clones with a single transgene copy were identified (called CLOSITRA for CLOnes with SIngle TRAnsgene) and all appeared to show varying β-glucuronidase activity. Thus, CLOSITRA 36, 40, 59, and 60 showed no β-glucuronidase activity (level 0), clone 33 medium activity (level 2), and clones 78 and 72 strong activity (respectively level 3 and 4). The clone genotypes are listed in Table 1. The three single-copy clones exhibiting β-glucuronidase activity appeared late during the kinetic analysis relative to the higher copy-number clones (Supplementary Figure S6).

**TABLE 1:** The phenotypic and genotypic characterization of single-copy transgene clones (CLOSITRA). For each clone, the level of GUS intensity relative to the GUS5-positive control (Row 3), the cassette integrity information measured after PCR analysis of the region surrounding the polycistronic cassette from the FcpB promoter to the FcpA terminator (Supplementary Figure S9) (Row 4), and the result for the amplicon (Row 5) are indicated.

Constructs		NAT-T2A-GUS					GUS-T2A-NAT		NAT-P2A-GUS		GUS-P2A-NAT
Clones	33	59	60	72	73	36	40	78	91	92	—
GUS Level	2	0	0	4	0	0	0	3	0	0	—
Cassette Integrity	✓	X	X	✓	X	✓	✓	✓	X	X	—
Sequence integrity	✓	X	X	✓	X	✓	✓	✓	X	X	—

Overall, our results show that there tends to be no correlation between transgene copy number and β-glucuronidase activity. However, there appears to be a higher probability of identifying CLOSITRA among clones with late-onset activity.

### 3.5 Genotypic Characterization of Clones With Single Transgenes

Droplet Digital PCR technology enabled us to identify 10 CLOSITRA within the NTG, NPG, and GTN groups. Genotypically, 80% harbored a transgene cassette in which the *uidA* gene was in the second position. Phenotypically, only 30% of this population was GUS (+), with one level 2 (clone 33), one level 3 (clone 78) and one level 4 (clone 72) (Table 1). We went one-step further in the molecular characterization to understand the lack of β-glucuronidase activity in 70% of the clones by sequencing the entire transgene cassette from the promoter to the terminator. PCR analysis showed the three GUS (+) CLOSITRA to have amplicons of the expected size (approximately 3,200 bp). Among the GUS (−) CLOSITRA, five of seven (59, 60, 73, 91 and 92) showed a smaller amplicon (Supplementary Figure S9). The sequencing of these five clones showed large deletions in the *uidA* sequence (data not shown). These clones were NAT-resistant, as the *NAT* gene was full length, without β-glucuronidase activity due to truncation of the *uidA* gene. Intriguingly, CLOSITRA 36 and 40 did not carry any transgene sequence modifications to explain their GUS (−) phenotype.

We wished to determine whether clones 36 and 40 show no activity or weak non-detectable activity. We thus developed a fluorometric assay based on the cleavage of 4-methylumbelliferyl-β-D-glucuronide (MUG), a fluorogenic substrate, by β-glucuronidase (Mead et al., 1955). Upon cleavage, the fluorescent moiety 4-methylumbelliferyl (4-MU) is released and detected by excitation (approximately 360 nm). Ten-hour kinetic monitoring of the fluorescence in GUS (+) CLOSITRA protein extracts was performed.

**FIGURE 5:**
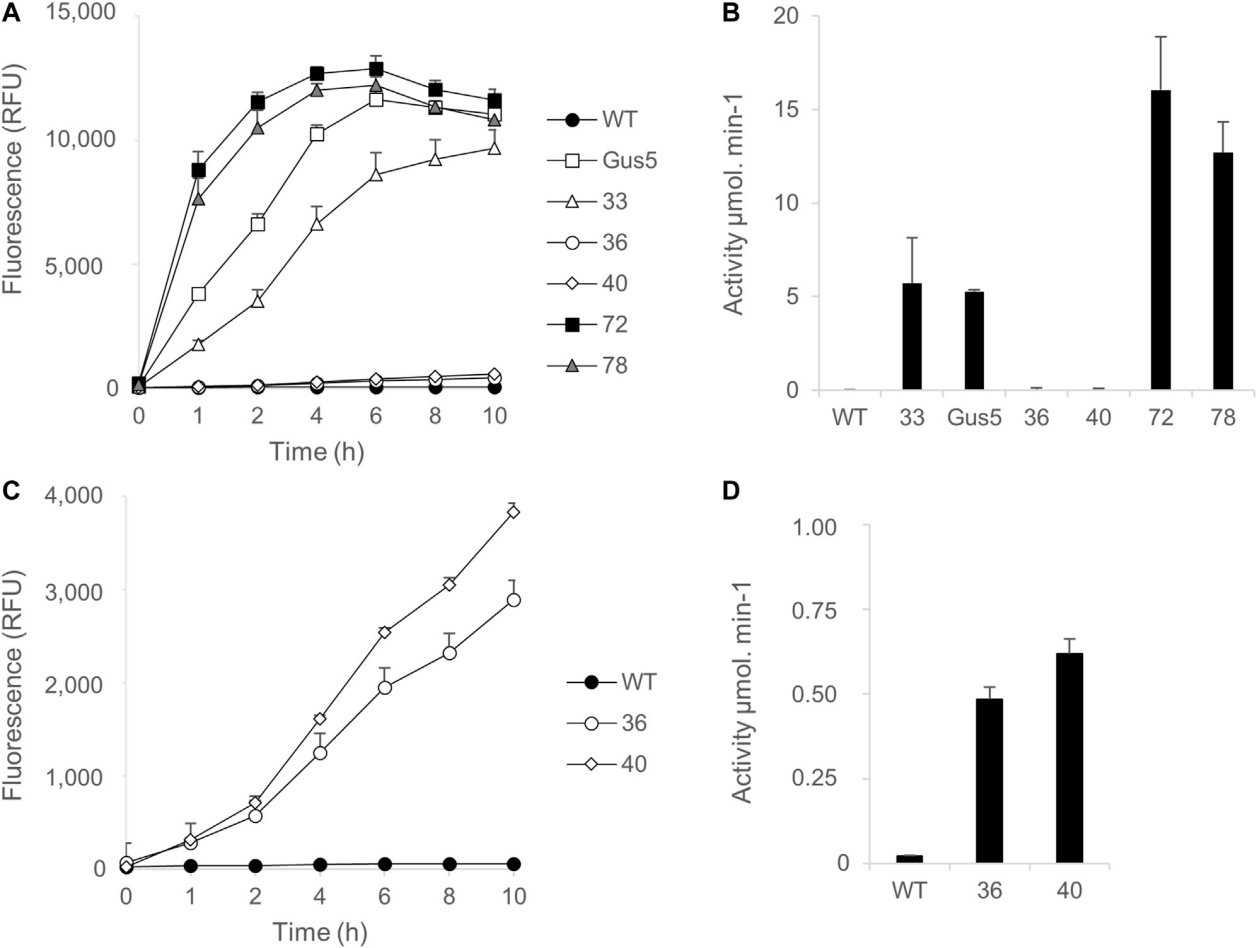
Quantification of β-glucuronidase activity in single-copy transgene clones (CLOSITRA) using synthetic fluorogenic substrates. **(A)** Fluorescence [relative Fluorescence Unit (RFU)] measured in all clones harboring an intact single-copy transgene using 1 µM of the substrate MUG. β -glucuronidase activity was measured by the accumulation of the fluorogenic glucuronidase product 4-methylumbelliferone (4-MU) and is represented as relative fluorescence units (RFU) on the *y*-axis. WT lysate was included as a negative control and Gus5 as the reference strain and positive control. Data points represent the average of triplicate technical measurements. Error bars represent standard deviation (SD) of the means of two independent experiments. **(B)** Glucuronidase activity (µmol.min^−1^) was measured after 1 h of incubation in the presence of 1 µM of the substrate MUG. **(C)** Fluorescence [relative Fluorescence Unit (RFU)] measured for clones 36 and 40 using 10 µM of the substrate MUG. (**D**) Glucuronidase activity (µmol.min^−1^) was measured after 4 h of incubation in presence of 10 µM of the substrate MUG.

First, kinetic monitoring was performed using 1 µM MUG substrate, with wild type and Gus5 strains added to the experiment as negative and positive controls, respectively (Figures 5A,C). All GUS (+) CLOSITRA showed β-glucuronidase activity (Figures 5B,D), characterized by an increase in fluorescence over time (Figure 5A). Both CLOSITRA 72 and 78 reached their maximal absorbance after 6 h, reaching 12,891 and 12,205 relative fluorescence units (RFU), respectively, against 11,642 for the reference strain Gus5. The β-glucuronidase activity was measured after 1 h of incubation with 1 µM of MUG substrate (Figure 5B). Both CLOSITRA 36 and 40 showed weak enzymatic activity. Although their activity was approximately 40 and 30 times lower than that of CLOSITRA 72 at its maximum (15 µmol.min^−1^), respectively, they still showed higher activity than the wild type control (Figure 5B). Kinetic monitoring with 10 µM MUG was performed on all five CLOSITRA to confirm this result. CLOSITRA 33, 72, and 78, as well as Gus5, reached saturating levels of fluorescence after 1 h. Both CLOSITRA 36 and 40 showed unquestionable activity (Figure 5D). After 4 h, the β-glucuronidase activity for clone 36 and 40 was 0.48 and 0.62 µmol.min^−1^, respectively (Figure 5D), whereas the activity was 0.02 µmol.min^−1^ for the wild type control. Therefore, these two clones show low β-glucuronidase activity that could not be detected by the colorimetric assay.

These results confirm that clone 72 had the highest activity, followed by clone 78 and then clones 33, 40, and 36. Clones 72 and 78 showed higher activity than clone Gus5, which has 22 copies of the *uidA* gene integrated. Although it is not possible to know how many of these copies are functional, this demonstrates that it is possible to identify clones with a single copy and high β-glucuronidase activity (Figure 5B).

Overall, these results demonstrate that a single copy of a transgene can drive varying gene expression, here a gene for NAT resistance and a β-glucuronidase gene. However, polycistronic expression does not systematically ensure double expression. All the clones showed an intact NAT-gene sequence, which was expected, as the clones were selected based on NAT-resistance. Nevertheless, polycistronic systems do not guarantee that NAT-resistant clones also express the transgene of interest, as 5 of 10 clones had truncated *uidA* sequences. Finally, the fact that the GUS (+) CLOSITRA clones showed three levels of intensity suggests that the insertion locus may have an impact on transgene expression.

### 3.6 Impact of the Integration Locus on Transgene Expression

**FIGURE 6:**
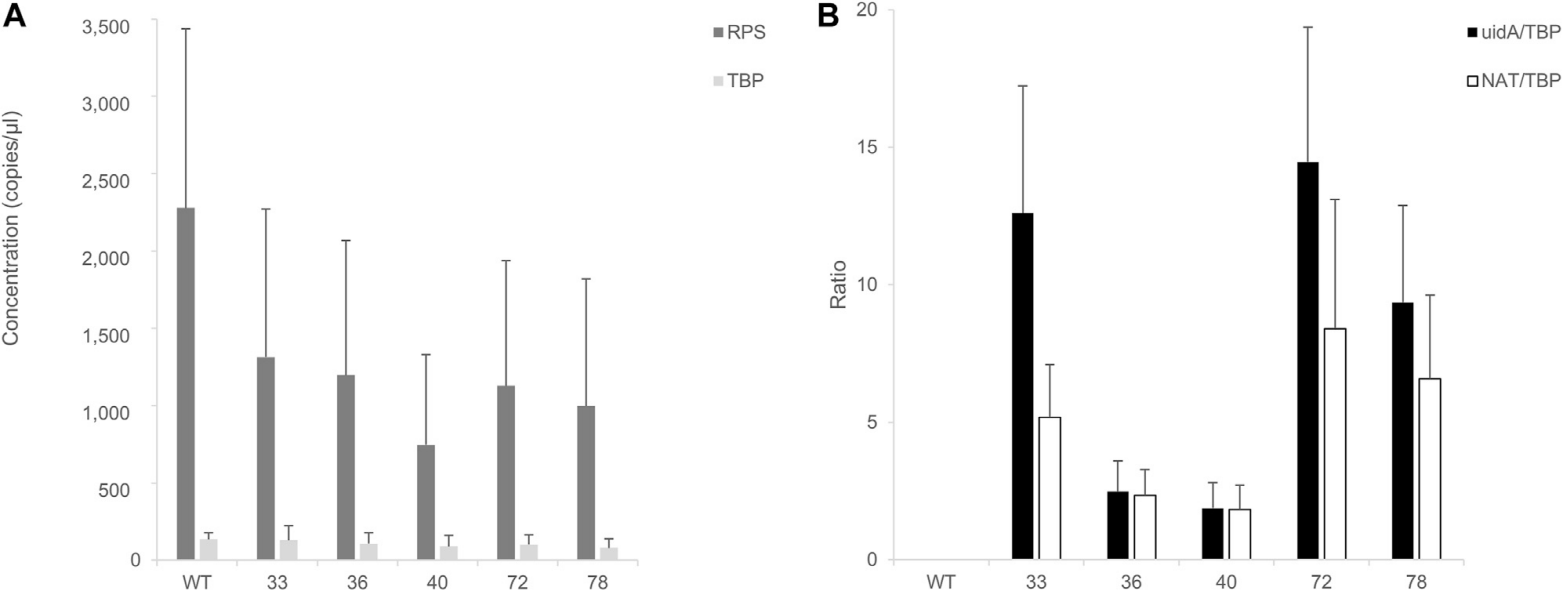
Assessment of RT-ddPCR for absolute mRNA quantification. **(A)** Quantification of *RPS* and *TBP* mRNA concentrations (copies/µl) in clones harboring a single and intact copy number of transgene (33, 36, 40, 72, 78). Bar chart of cDNA concentrations (*y*-axis) detected by ddPCR of *TBP* (light gray) and *RPS* (dark gray). Wild type (WT) is included as control. Error bars in figure represent standard deviation (SD) of the means of three independent experiments. **(B)** Quantification of *uidA* and *NAT* mRNA level. Normalized ratios of the cDNA concentration of targeted genes (*uidA*: black; *NAT*: white) on *TBP* concentrations detected in the clones are from three independent experiments. A wild type (WT) strain is included as a negative control. *uidA*/*TBP* ratios calculated for each clones 33, 72, and 78 were individually compared to clone 36 *uidA*/*TBP* ratio and clone 40 *uidA*/*TBP* ratio and were found statistically different (*t*-test, *p*-value < 0.05) (*uidA_33* vs. *uidA_40*, *p*-value = 0.01692; *uidA_33* vs. *uidA_36*, *p*-value = 0.02099; *uidA_36* vs. *uidA_72*, *p*-value = 0.01457; *uidA_40* vs. *uidA_72*, *p*-value = 0.01204; *uidA_36* vs. *uidA_78*, *p*-value = 0.03219; *uidA_40* vs. *uidA_78*, *p*-value = 0.0237).

Encouraged by these results, we aimed to study the impact of the insertion locus on transgene expression. RNA was extracted from GUS (+) CLOSITRA, purified, and subjected to reverse transcription. The generated cDNAs were used for absolute quantification by ddPCR. First, the mRNA levels of the two endogenous genes, *RPS* and *TBP*, in GUS (+) CLOSITRA were quantified (Figure 6). Overall, the concentration of *TBP* was similar in all clones, with values of 150–220 copies/µl. Although the concentration of *RPS* was also similar in our samples, it was at least 10-fold higher, with values of approximately 1,060 to 2,700 copies/µl. Thus, the *RPS* gene is much more highly expressed than the *TBP* gene (Supplementary Figure S10). This confirms the results obtained in a previous study, which showed that *RPS* shows transcript levels about 10 times higher than those of *TBP* by q-PCR analysis (Siaut et al., 2007). We used *TBP* as the reference gene for the subsequent experiments to avoid saturation. We next compared *uidA* and *NAT* transcript levels in all CLOSITRA (Figure 6). The concentration of the targeted gene was normalized according to the concentration of the *TBP* reference gene. As expected, clones 33, 72 and 78 show the highest *uidA* transcript levels compared to clones 36 and 40 (*p*-value <0.05, see exact *p*-value in the legend of the Figure 6). Intriguingly, clone 33 exhibits a reproducible discrepancy in the level of *uidA* and *NAT* transcripts.

These results show that the insertion locus plays a major role in transgene expression. It was thus imperative to sequence the insertion loci to increase our understanding and open the way to manipulate *P. tricornutum* for better characteristics.

### 3.7 Identification of Transgene Integration Sites

*Phaeodactylum tricornutum* is a diploid organism, for which the reference genome was originally assembled as 33 chromosome-sized scaffolds (Bowler et al., 2008). We precisely determined the transgene integration sites within CLOSITRA 33, 36, 40, 72, and 78 by MinION sequencing. After *de novo* assembly, the contigs harboring the plasmid elements were identified by alignment for each clone. All displayed at least 45% percent coverage and three had 99% identity with the transformation vector. Four different integration sites have been identified for CLOSITRA 33, 36, 72 and 78 (Table 2). Both clones 36 and 40 share the same insertion locus, which suggests they are identical. The constructs were integrated into chromosomes 4, 5, 1, and 6 in CLOSITRA 33, 36, 72, and 78 respectively, corresponding to the six largest chromosomes of the genome of *P. tricornutum*. Moreover, plasmid integration led to gene disruption in CLOSITRA 33, 36, and 78. In CLOSITRA 72, the vector was located within a small intergenic region (less than 1 kb) (Table 2). However, no noticeable difference was observed on the growth of strains 72, 78, and 33 compared to the wild type strain (Supplementary Figure S11), demonstrating that close gene integration does not affect the viability of strains. Further analyses are ongoing to understand the integration pattern of the transformation vector and measure the impact of transgene insertion on the neighboring genes.

**TABLE 2:** Summarized details of integration sites of clones harboring a functional single-copy integrated transgene.

Clone	Chromosome	Position (bp)	Genetic feature	AA size	Upstream feature	Downstream feature
33	4	1,348,875–1,349,990	PHATRDRAFT_34196 (single exon coding protein)	371 aa	Intergenic region	Intergenic region
36/40	5	770,738–773,743	PHATR3_EG01314.t1 (single exon protein coding gene)	1,001 aa	Phatr3_J54330.t1	Intergenic region
72	1	715,346–715,811	Intergenic region	NA	PHATR3_J42656.t1	PHATR3_J9639.t1
78	6	465,049–466,776	Phatr3_J45200.t1 (single exon protein coding gene)	575 aa	Phatr3_EG02107.t1	Phatr3_EG01392.t1

## 4 Discussion

Several studies have reported that clones resulting from random integration result in heterogeneous transgene expression in mammals, plants (Butaye et al., 2005; Daboussi et al., 2012; Laboulaye et al., 2018), and, more recently, diatoms (George et al., 2020). Although chromosomal context has often been claimed to be responsible for such heterogeneity, factors such as copy number, methylation, and chromatin context, have been rarely investigated (Fagard and Vaucheret, 2000; Daboussi et al., 2012; Alhaji et al., 2019). Here, we conducted an exhaustive analysis in which 93 clones were both genetically and phenotypically characterized. This was made possible by the development of a pipeline integrating state-of-the-art technologies and higher throughput than traditional methods. Such a pipeline reduced the screening time and costs. This is the first report to systematically analyze the correlation between copy number and enzyme activity in diatoms (Supplementary Figures S12–S15). This study demonstrates that high expression in clones is generally associated with the integration of multiple transgenes and, furthermore, highlights the danger of selecting transgenic strains based solely on the production of a specific molecule when the number of integrated transgenes is very large. Indeed, this increases the probability of collateral damage, such as disruption or modification of the expression of genes close to the integration site.

Analysis of CLOSITRA clones, harboring a single integrated copy, showed that some exhibit transgene expression equivalent to that measured in clones with multiple integrated copies. This analysis also showed that more than 50% of the CLOSITRA clones had a non-functional (disrupted) single-integration cassette. Furthermore, sequencing of these clones showed that all CLOSITRA with β-glucuronidase activity had a transgene integration site in the vicinity of other genes, which is not surprising due to the very compact genome of diatoms.

To determine whether the identified loci can be considered as potential safe habors (Sadelain et al., 2011), it will be necessary to first determine the impact of transgene integration on neighboring genes and, second, to measure the stability of the transgene during the semi-industrial scale production process, in which cells are subjected to various limitations and stresses (pH, pressure, substrate gradients, etc.). Once these loci are validated, it will then be possible to create a landing pad containing a recombination site and selectable marker to ensure efficient and predictable transgene integration.

A key question remains as to whether the transgene can be maintained without additional selective pressure. Several reports have shown that transgene expression decreases over time *in vivo* due to promoter methylation and loss of the transgene copy (Migliaccio et al., 2000). Here, we observed no change in β-glucuronidase activity over the 2 years of the project for clones with a single integrated copy (78, 72, and 33). In addition, individual subclones of CLOSITRA 72 were maintained independently in the presence or absence of selection to estimate the probability of losing the transgene. The clones were resistant whether or not they were grown long-term in the presence of NAT (data not shown). This result is promising as it shows that the transgene integration at this site does not affect cellular fitness of *Phaeodactylum tricornutum*. However, this result, monitored at the laboratory scale in a 96-well plate, should be evaluated in a bioreactor. In addition, selective pressure should be replaced with a more industry-compatible marker by evaluating auxotrophic markers. Such markers have recently been described by Serif et al. (2018) and Slattery (2020).

The identification of integration loci is a key issue for both basic and applied research. Many studies aiming to compare the strength of various promoters and various sequences do so in a context in which neither the copy number nor the integration locus is analyzed. By keeping the chromosomal environmental context constant, the integration of different constructs at the same locus should allow undeniable gains in terms of robustness and reliability of results. The use of diatoms as microbial cell factories for the production of compounds, such as antibodies, bioplastics, and terpenes, is an emerging field (Butler et al., 2020). The identification of “safe harbor” integration loci that enable stable and predictable transgene expression, without affecting that of neighboring genes, will be an important for improving strain stability and meeting industrial specifications. Thus, the AAVS1, Rosa26, CCR5 loci of CHO cells are used for the production of several molecules (Pavani and Amendola, 2021).

Although the first promising loci have been identified in this study, there is still a long way to go before such regions can be targeted. Indeed, it is necessary to introduce the selected constructs in the chosen loci while also avoiding random integration of other copies. Several studies have shown site-specific integration by creating a double stranded break mediated by molecular scissors (Weyman et al., 2015; Moosburner et al., 2020). Thus, Moosburner et al. obtained six clones with a cassette inserted at the target locus exhibiting the expected phenotype. However, only one had an insertion at both alleles, which represents a 17% yield for the production of bi-allelic mutants by HR (Moosburner et al., 2020). However, the presence of randomly integrated supernumerary copies, intact or not, has not been analyzed. It is highly probable that targeted gene insertion is generally accompanied by random integration of a large number of copies. It will therefore be necessary to screen a large number of clones and/or to modify the balance between homologous recombination and random integration to increase the ratio of targeted to random integration.

In summary, the integrated pipeline has enabled the identification of stable loci for high-level heterologous gene expression. This work paves the way towards the development of *Phaeodactylum tricornutum* as efficient and robust microbial cell factories.

## Data Availability

The datasets presented in this study can be found in online repositories. The names of the repository/repositories and accession number(s) can be found in the article. The datasets generated for this study can be found in https://www.ncbi.nlm.nih.gov/Traces/study/?acc=PRJNA743167.
