# Supplementary material for: Identification of Loci Enabling Stable and High-Level Heterologous Gene Expression
Source: Front Bioeng Biotechnol. 2021 Oct 1;9:734902. doi: 10.3389/fbioe.2021.734902 (PMC8517075; doi:10.3389/fbioe.2021.734902)
Supplement: Supplementary file 1 [file DataSheet1.pdf]

Supplementary Material

Defrel *et al*

A

B

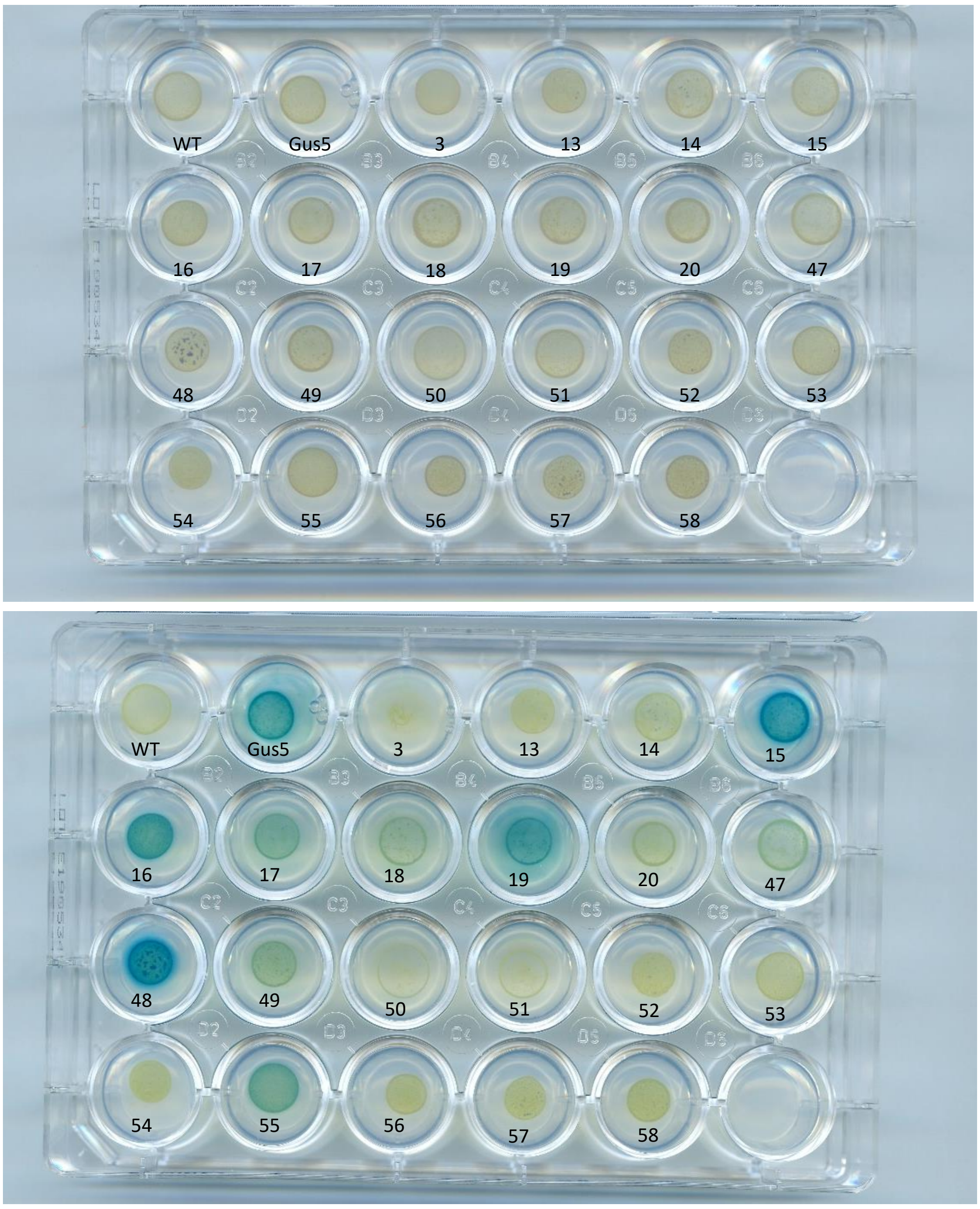

Supplementary Figure 1. Colorimetric β-glucuronidase assay in 24-well plate on group GUS-P2A-NAT. **(A)** Photo taken before addition of the reaction buffer. **(B)** Photo taken after 24 h of incubation at 37°C. The Gus5 strain was used as a positive control and both wild type (WT) and clone 3 (NAT transformation control) as negative controls. The names of the clones are indicated in the wells.

A

B

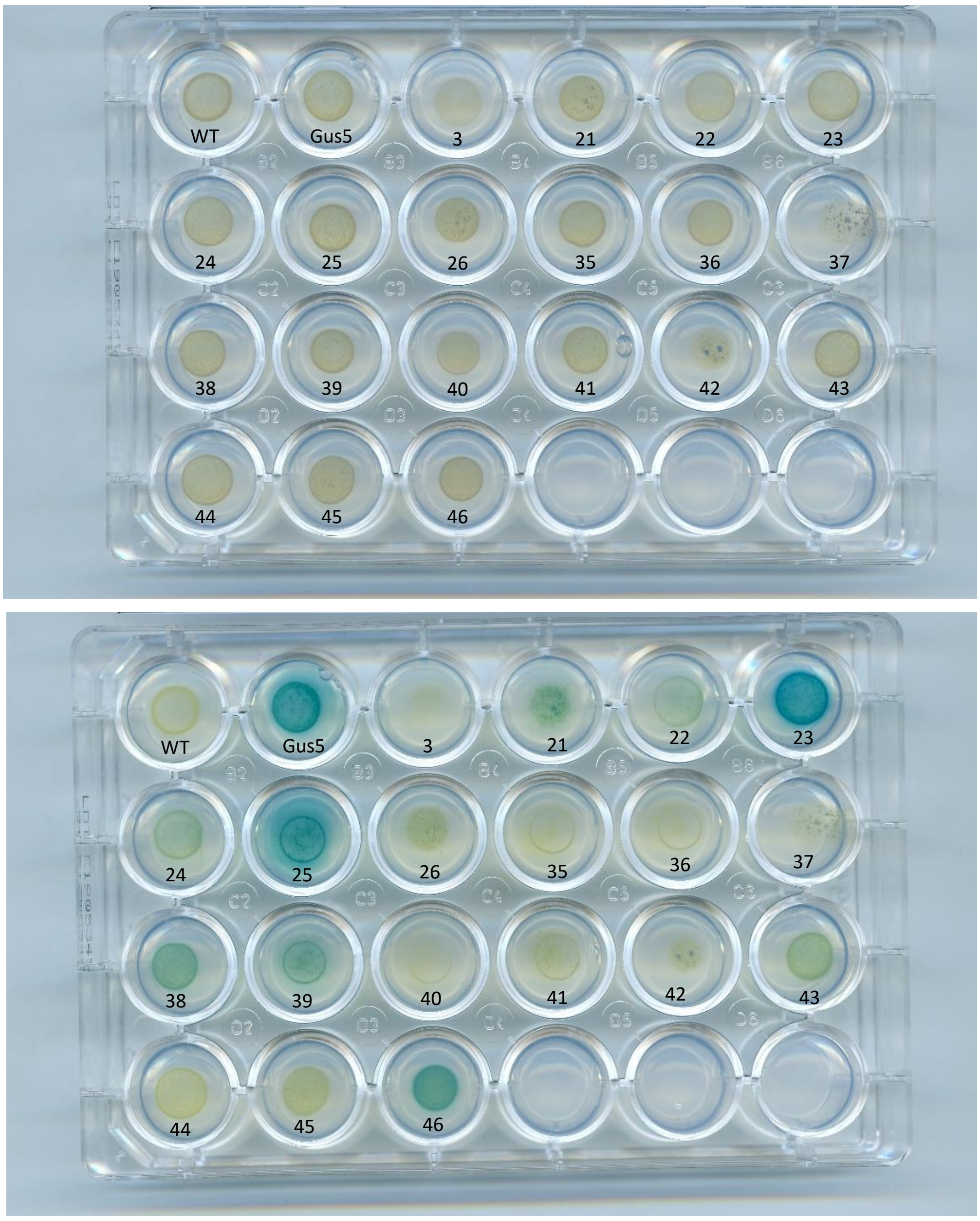

Supplementary Figure 2. Colorimetric β-glucuronidase assay in 24-well plate on group GUS-T2A-NAT. **(A)** Photo taken before addition of the reaction buffer. **(B)** Photo taken after 24 h of incubation at 37°C. The Gus5 strain was used as a positive control and both wild type (WT) and clone 3 (NAT transformation control) as negative controls. The names of the clones are indicated in the wells.

A

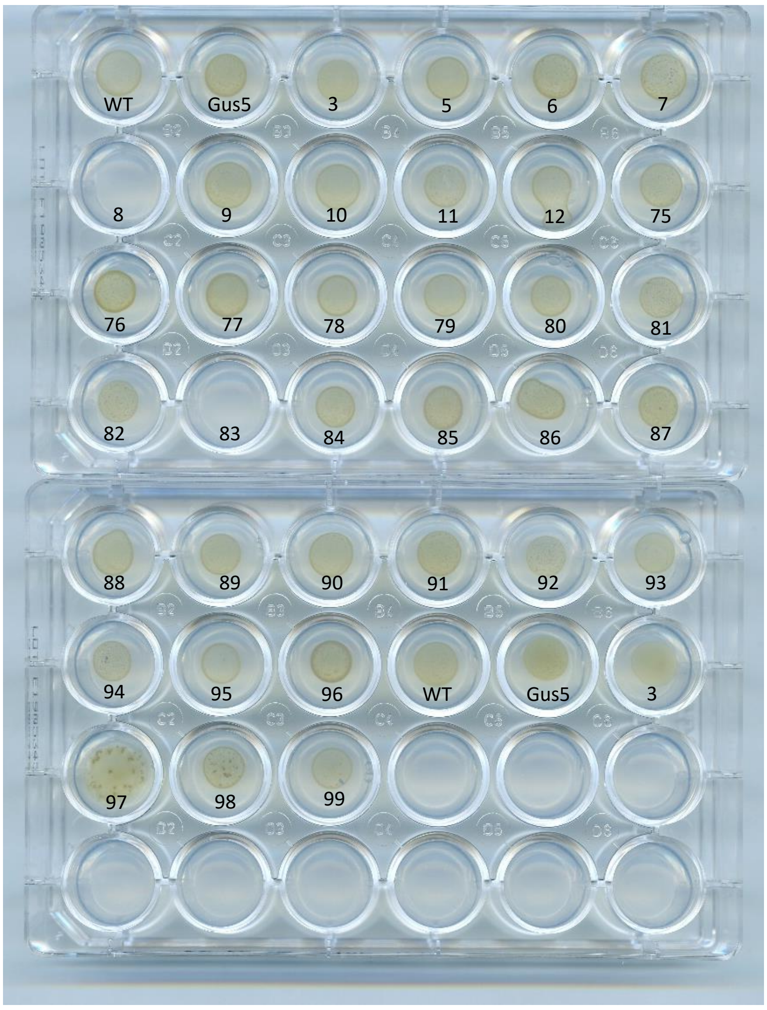

B

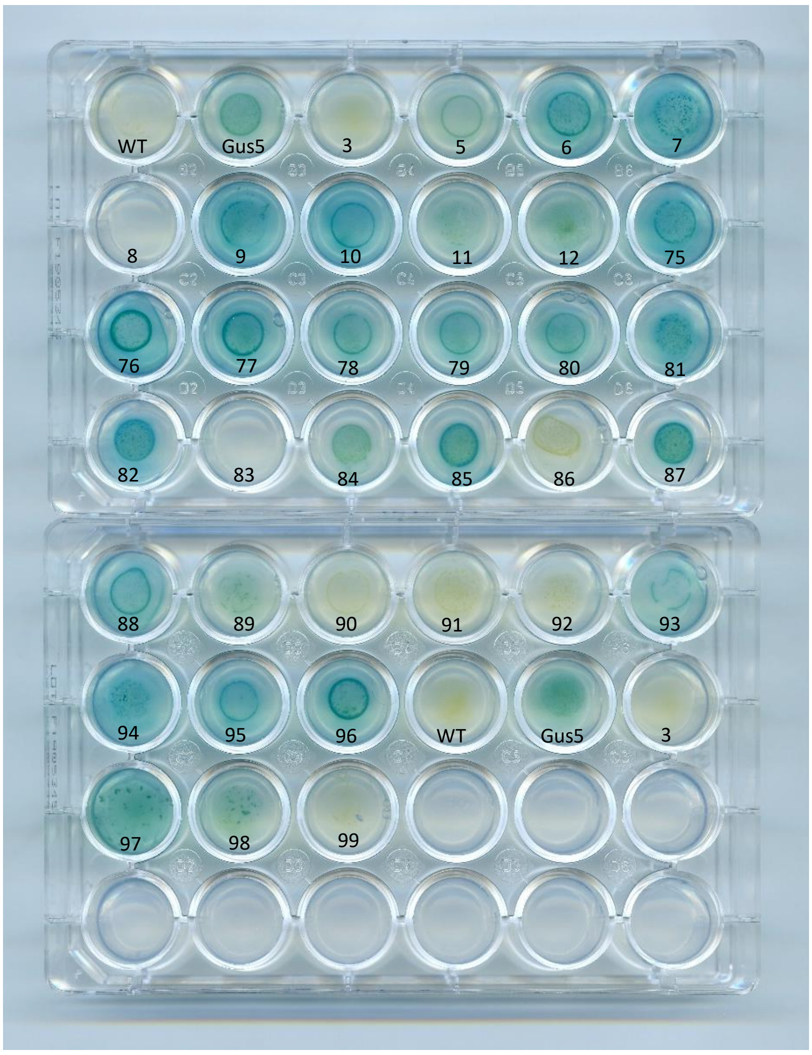

Supplementary Figure 3. Colorimetric β-glucuronidase assay in 24-well plate on group NAT-P2A-GUS. **(A)** Photo taken before addition of the reaction buffer. **(B)** Photo taken after 24 h of incubation at 37°C. The Gus5 strain was used as a positive control and both wild type (WT) and clone 3 (NAT transformation control) as negative controls. The names of the clones are indicated in the wells.

A

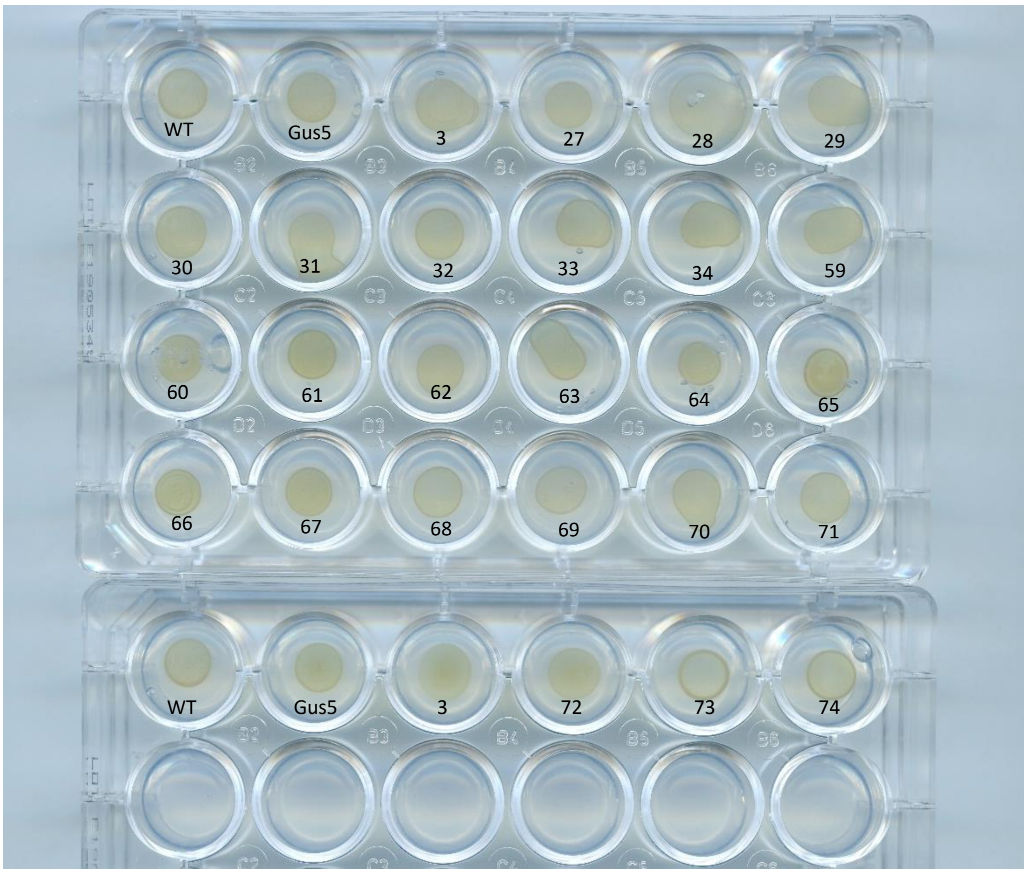

B

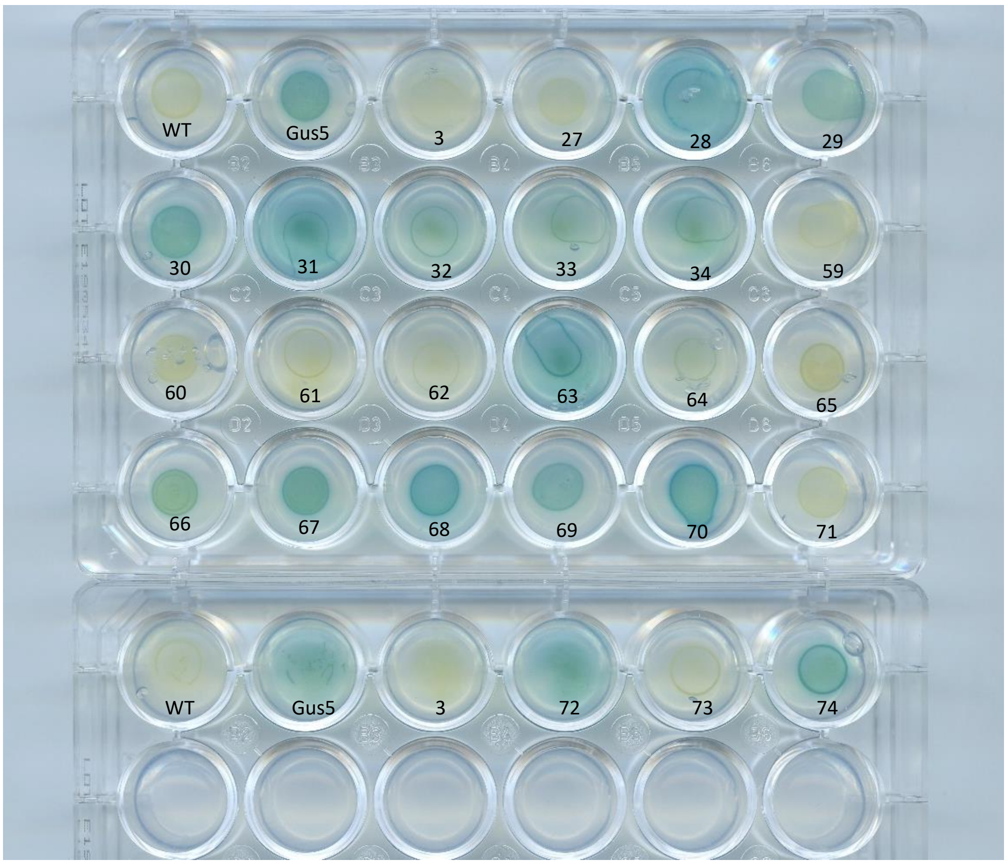

Supplementary Figure 4. Colorimetric β-glucuronidase assay in 24-well plate on group NAT-T2A-GUS. **(A)** Photo taken before addition of the reaction buffer. **(B)** Photo taken after 24 h of incubation at 37°C. Gus5 strain was used as a positive control and both wild type (WT) and clone 3 (NAT transformation control) as negative controls. The names of the clones are indicated in the wells.

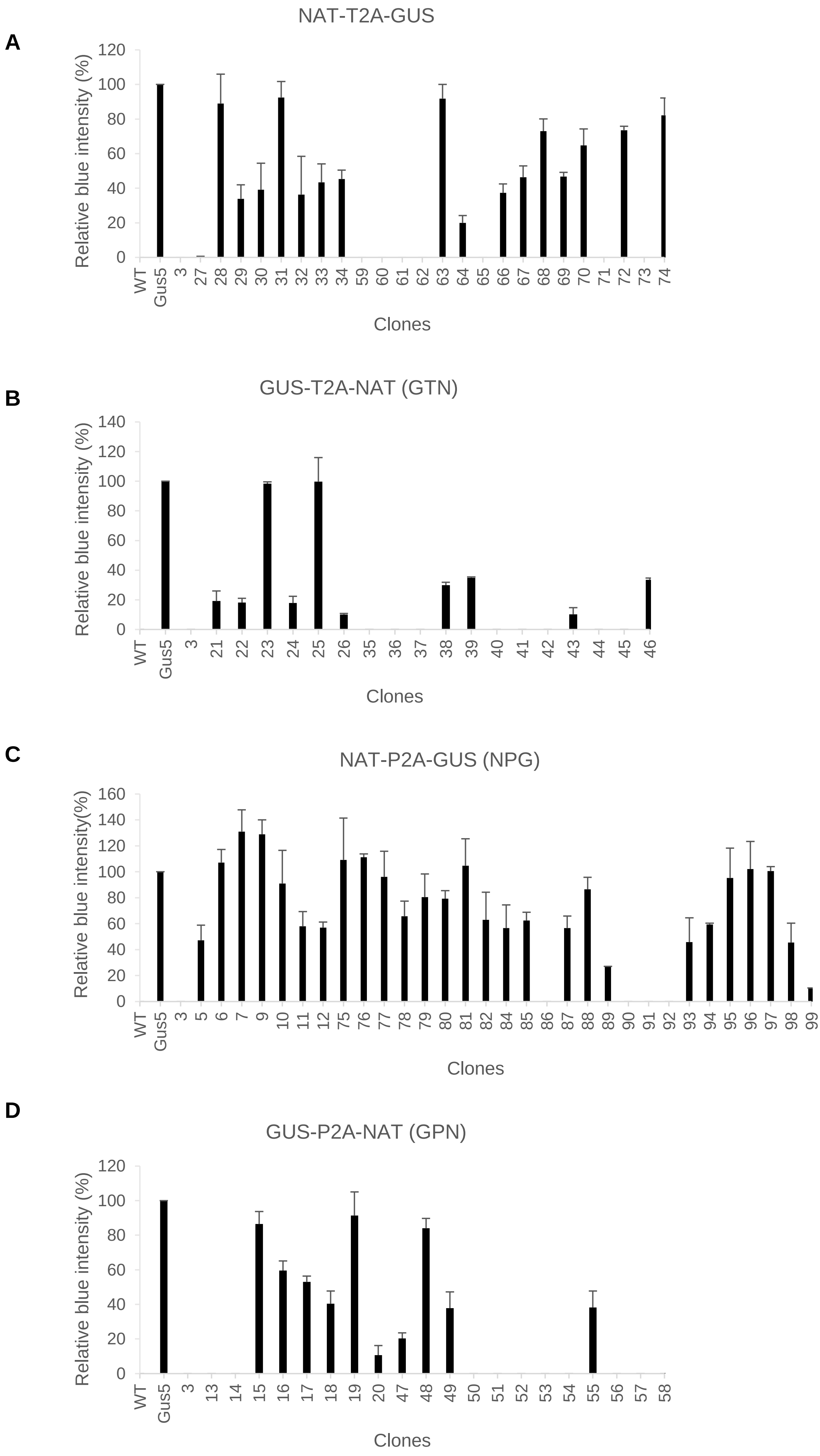

Supplementary Figure 5. Bar chart of relative color intensities calculated in groups at 24 h (Means and standard deviations were calculated from 2 biological replicates). **(A)** NAT-T2A-GUS. **(B)** GUS-T2A-NAT. **(C)** NAT-P2A-GUS, and **(D)** GUS-P2A-NAT. Gus5, as the referent strain was given a value of 100% and wild type (WT) and clone 3, a *NAT* transformation control (FcpB-NAT-FcpA), a value of 0%.

Supplementary Figure 6.

A

T=0h

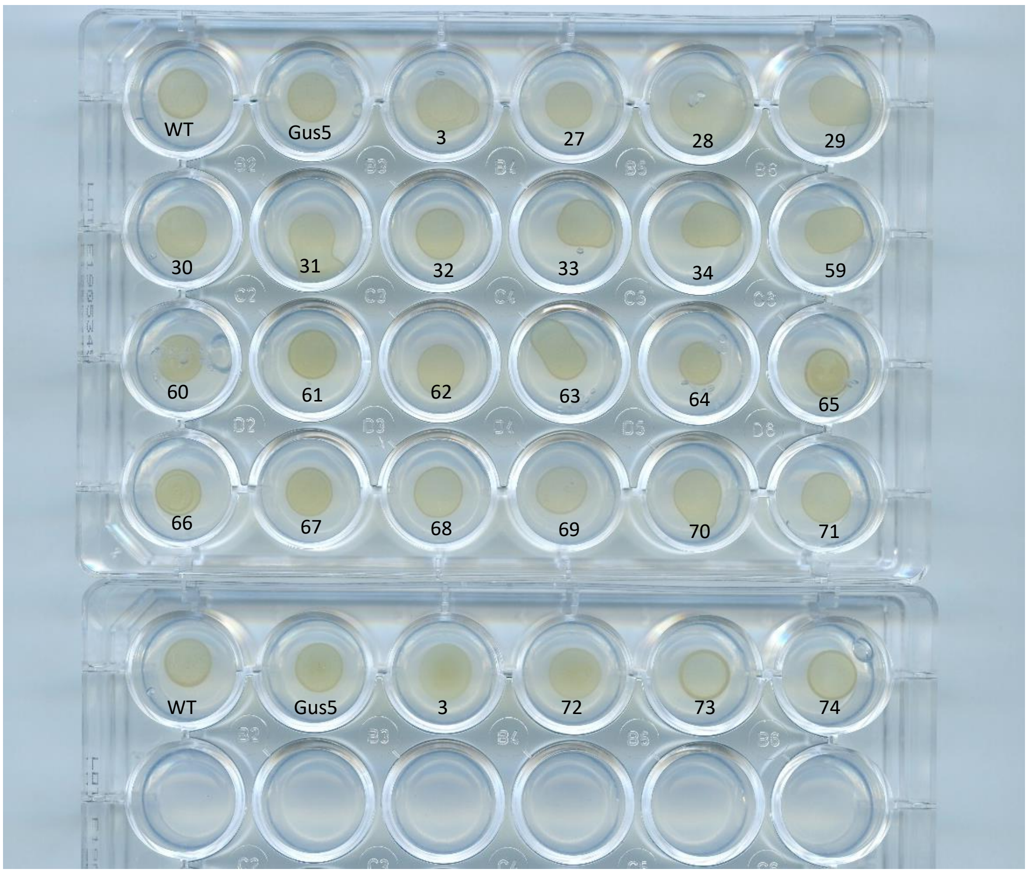

B

T=2h

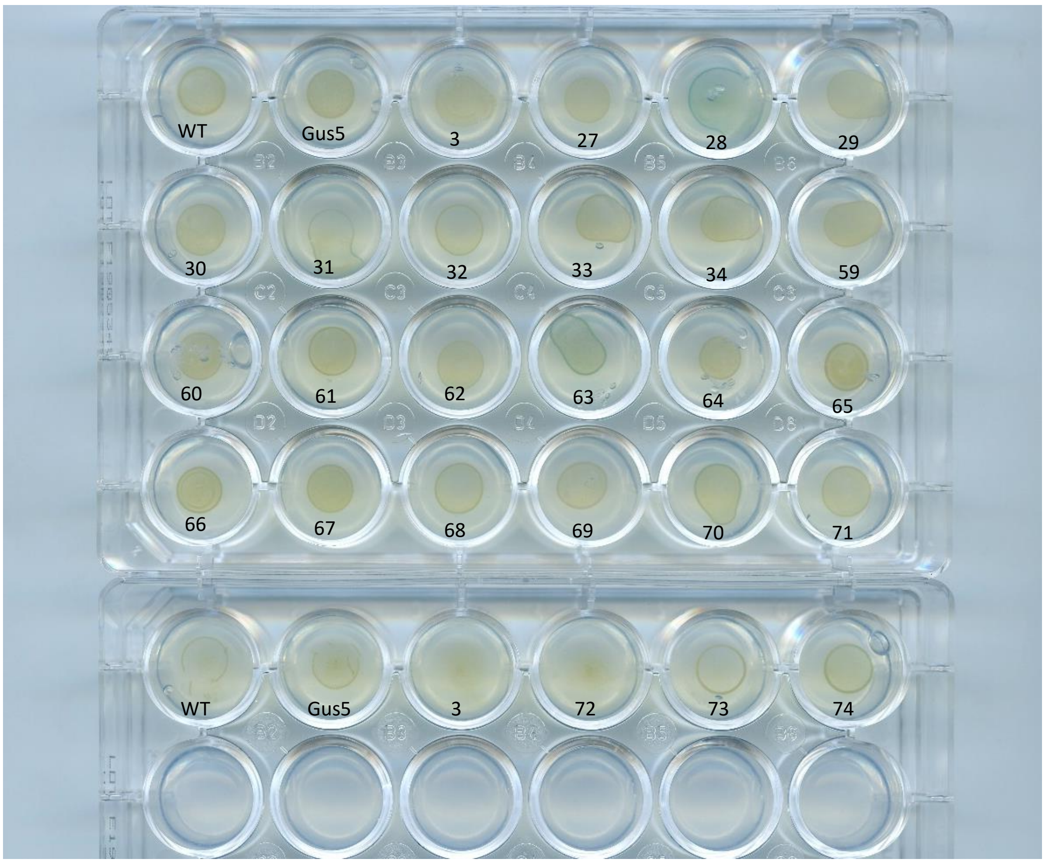

Supplementary Figure 6.

C

T=4h

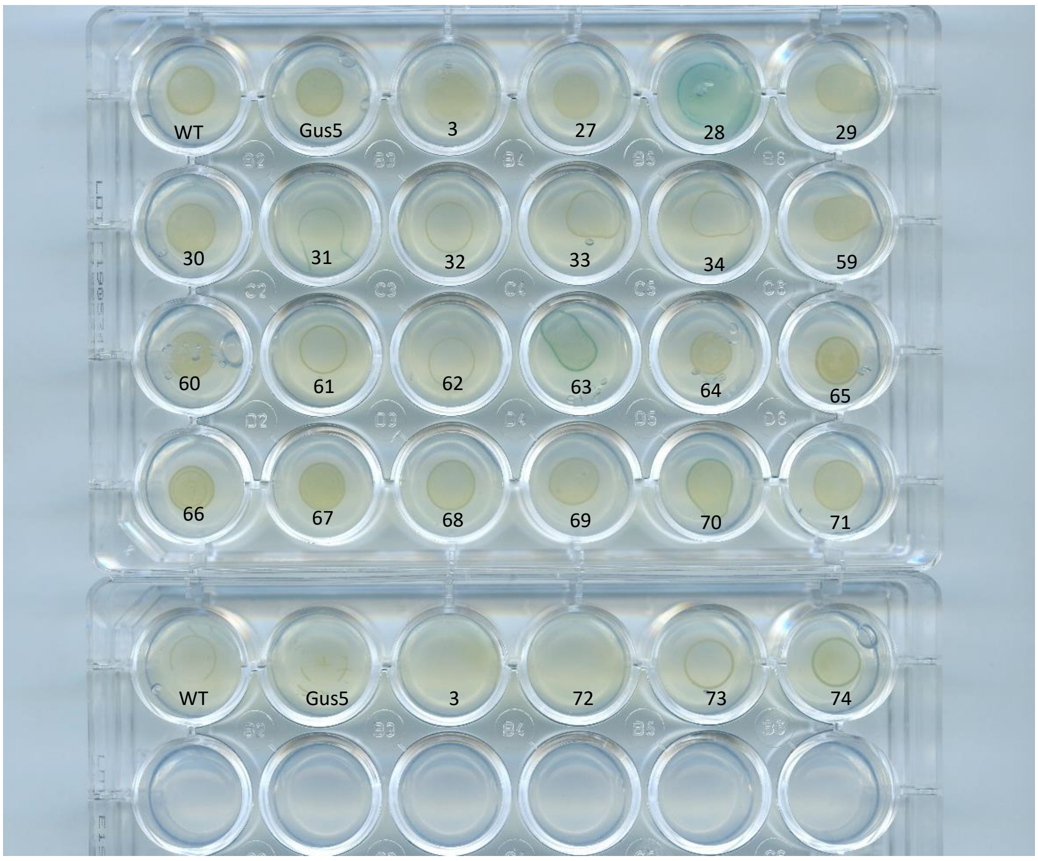

D

T=6h

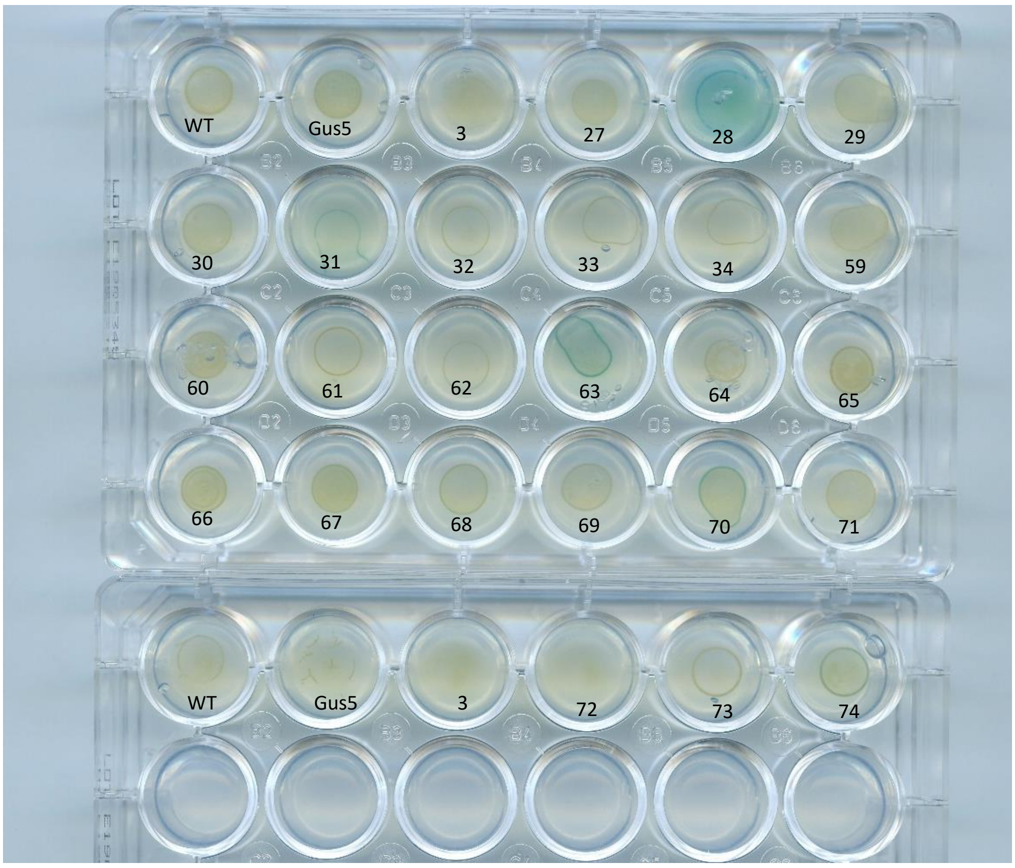

Supplementary Figure 6.

E

T=8h

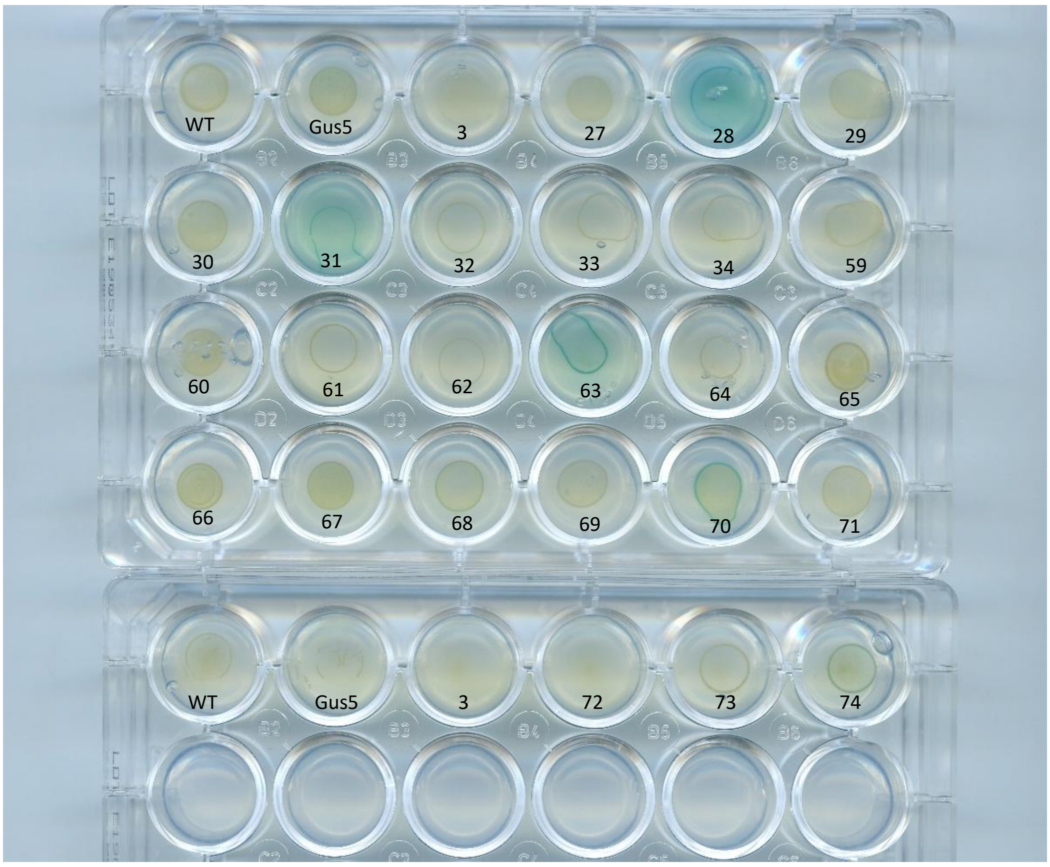

F

T=10h

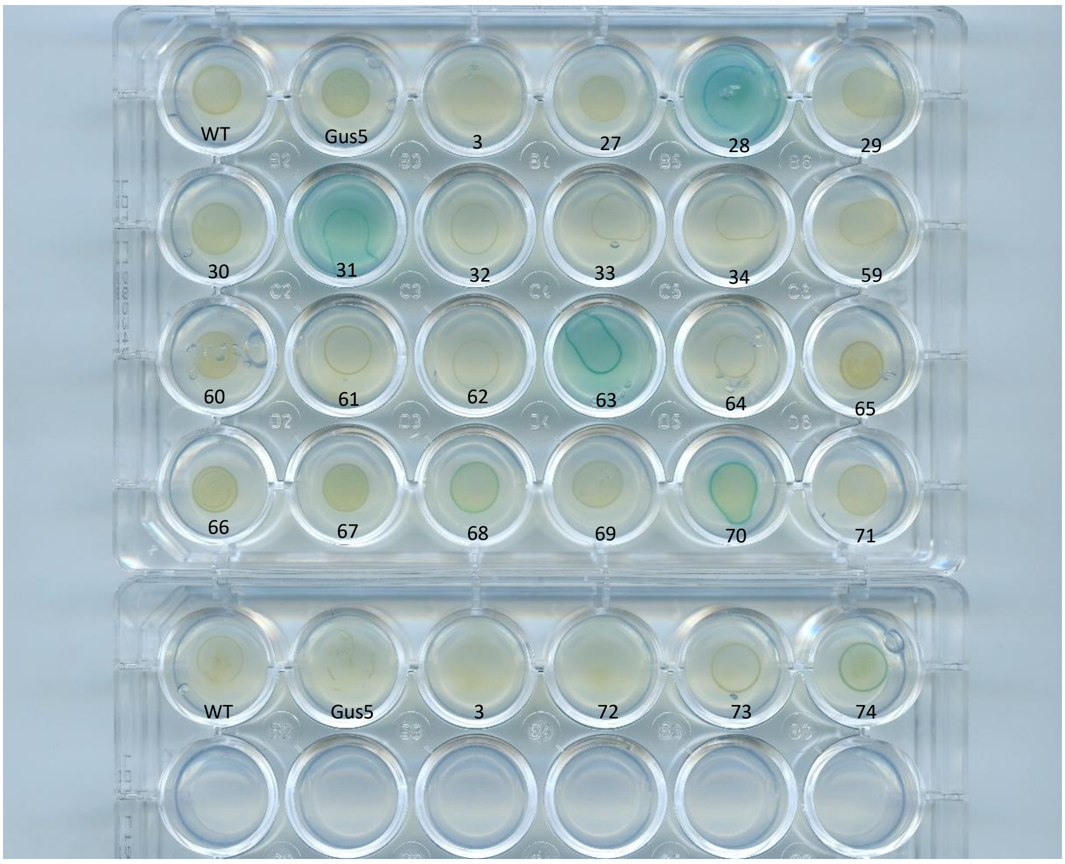

G

T=24h

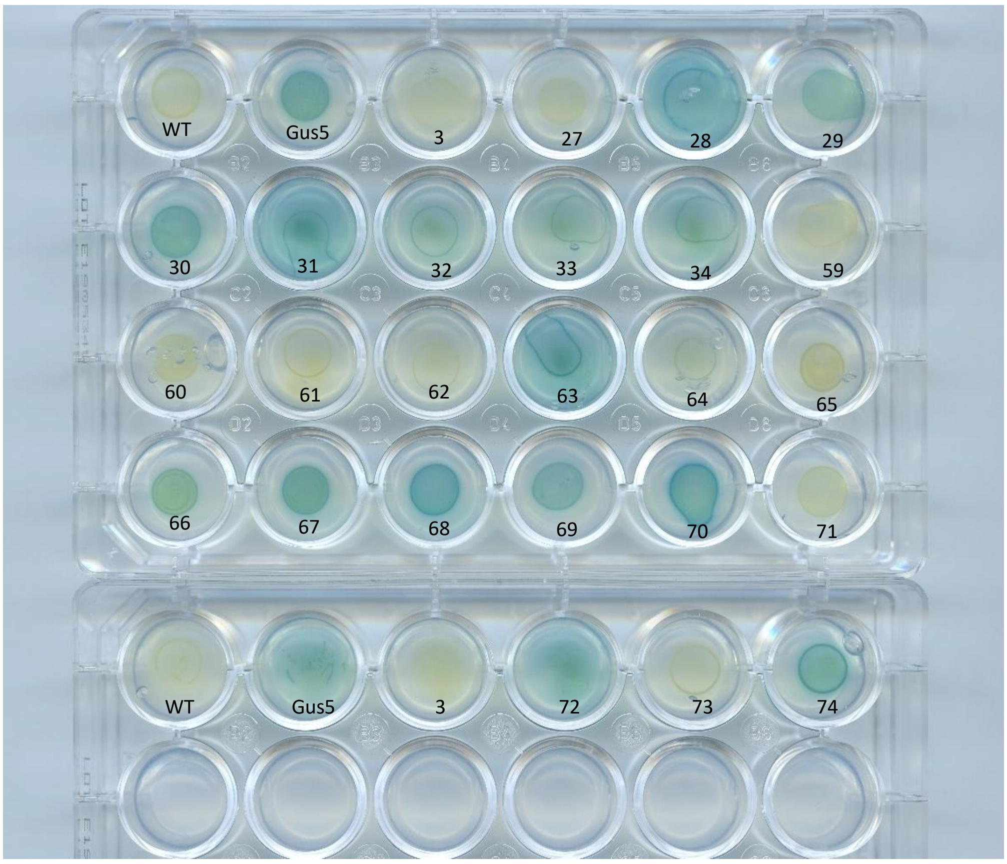

Supplementary Figure 6. Kinetics of colorimetric β-glucuronidase assay in 24-well plate on group NAT-T2A-GUS. **(A)** Photo taken before addition of the reaction buffer. **(B)** Photo taken after 2 h of incubation at 37°C. **(C)** Photo taken after 4 h of incubation at 37°C. **(D)** Photo taken after 6 h of incubation at 37°C. **(E)** Photo taken after 8 h of incubation at 37°C. **(F)** Photo taken after 10 h of incubation at 37°C. **(G)** Photo taken after 24 h of incubation at 37°C. Gus5 strain was used as a positive control and both wild type (WT) and clone 3 (NAT transformation control) as negative controls. Clone names are indicated in the well.

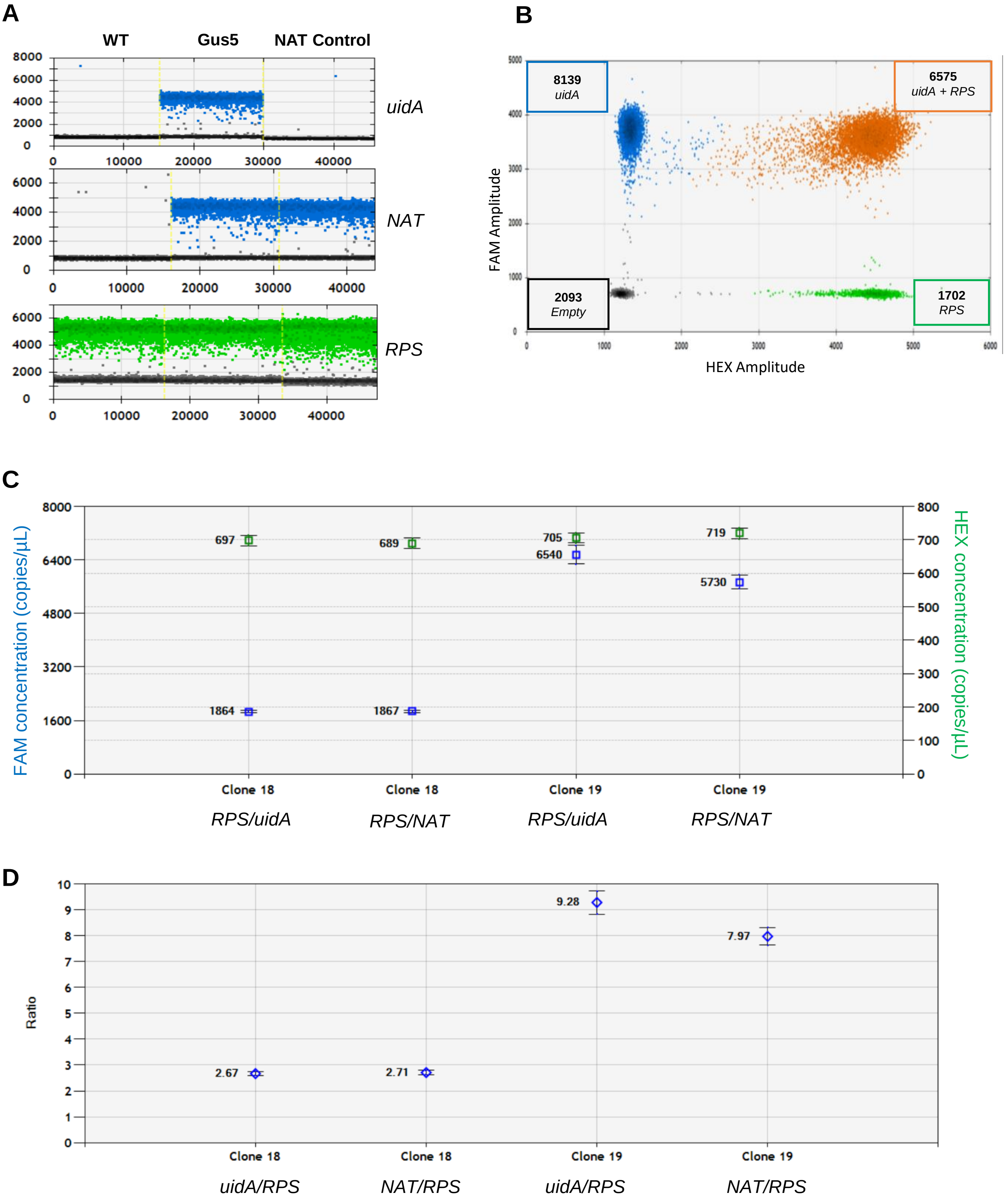

Supplementary Figure 7. Droplet Digital PCR (ddPCR): a robust technology to determine transgene copy number. **(A)** One-dimensional plot of droplets measured for fluorescence signal (amplitude indicated on y-axis) emitted from the endogenous reference gene *RPS* (HEX™-labelled, positive droplets are green) or the target genes *uidA* or *NAT* (FAM™-labelled, positive droplets are blue). Wildtype (WT), the Gus5 strain, and clone 3 (NAT transformation control) ere used as controls. Negative droplets are shown in black. **(B)** Two dimensional plot of droplets measured in clone 18 (GPN). Droplets containing both fluorescent probes are shown in red and negative droplets are shown in black. **(C)** Concentrations (copies/µL) measured from positive droplets for RPS (green) and *uidA* or *NAT* genes (blue) in clones 18 and 19**. (D)** Concentration ratios of target gene to reference gene (*uidA/RPS* or *NAT/RPS).* This ratio makes it possible to determine the transgene copy number. For example, there are 2.67 times more *uidA* copies in clone 18 than RPS. As *P. tricornutum* is diploid, there are thus 5.34 copies (2.67*2) of the *uidA* gene in clone 18.

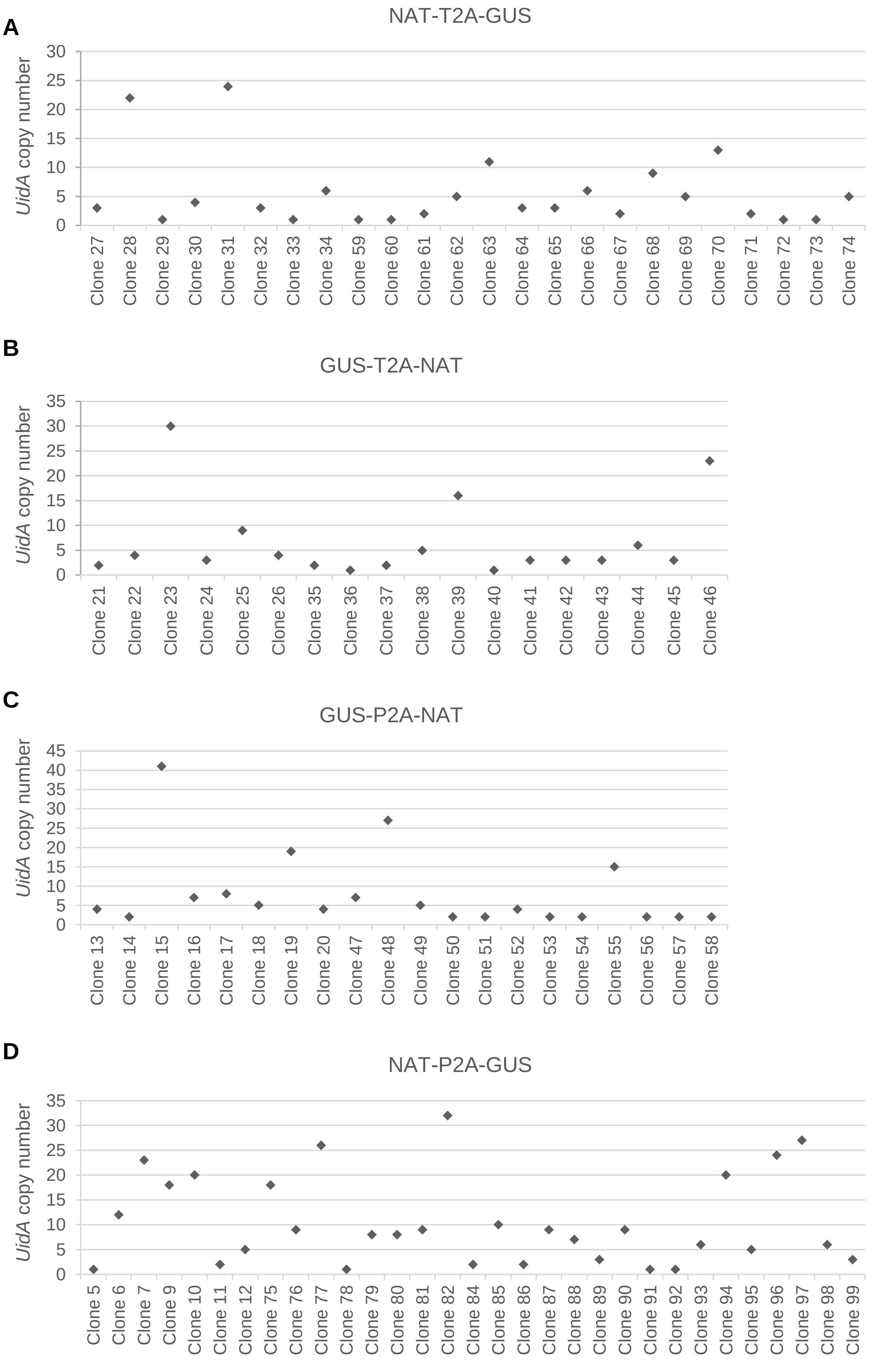

Supplementary Figure 8. Distribution of *uidA* copy number in the **(A)** NAT-T2A-GUS, **(B)** GUS-T2A-NAT, **(C)** GUS-P2A-NAT, and **(D)** NAT-P2A-GUS groups.

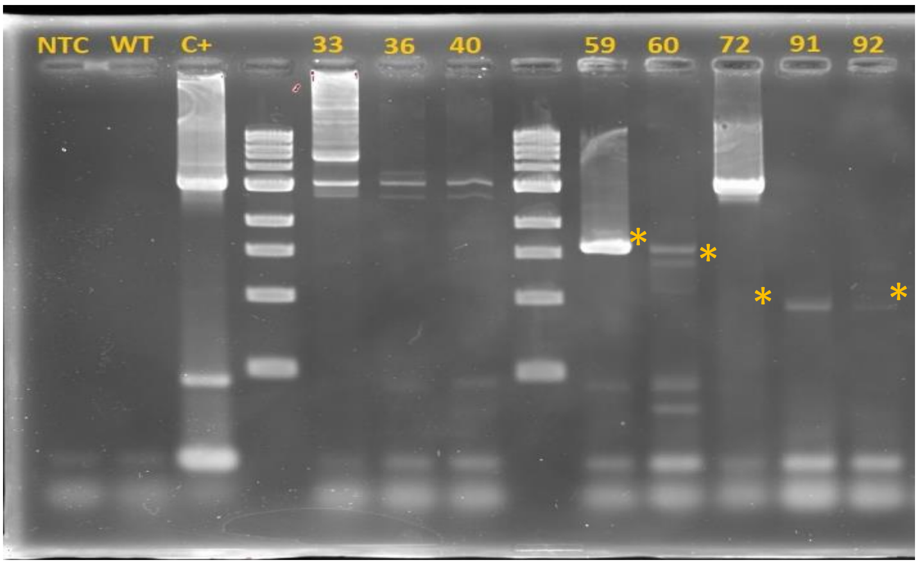

Supplementary Figure 9. Amplification of the transgene cassette in single-copy integrated clones. The 3,200 bp full-length amplicon is observed for clones 33, 36, 40, and 72. Truncated amplicons are observed for clones 59, 60, 91, 92 and indicated by a star (*). Purified plasmid (C+) and wildtype are included as positive and negative controls, respectively.

A

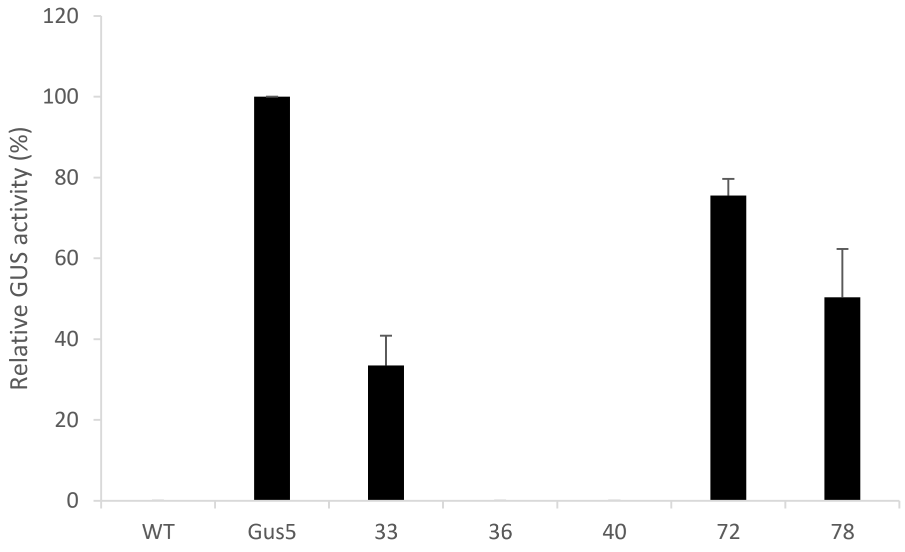

B

	Experiment	WT	Gus5	33	36	40	72	78
***uidA* copy number**	# 1	0	23.89	1.26	0.91	0.93	1.02	0.95
	# 2	0	21.72	1.28	ND	ND	1.01	0.95
	# 3	0	22.64	1.28	ND	ND	1.06	ND
	M	0	22.75	1.27	0.91	0.93	1.03	0.95
	SD	0	1.1	0.01	ND	ND	0.03	0.00

	Experiment	WT	Gus5	33	36	40	72	78
***NAT* copy number**	# 1	0	32.601	0.956	0.92	0.93	1.03	0.979
	# 2	0	36.142	1.036	ND	ND	1.015	0.93
	# 3	0	31.579	1.055	ND	ND	1.01	ND
	M	0	33.44	1.02	0.92	0.93	1.02	0.95
	SD	0	2.39	0.05	ND	ND	0.01	0.03

M = mean, SD = standard deviation

Supplementary Figure 10. Phenotypic and molecular characterization of single-copy transgenes. (A) Bar chart of relative color intensities calculated in clones 33, 36, 40, 72, and 78 at 24 h. Values are presented as the average of relative color intensities at 24 h for three independent colorimetric experiments. Gus5 and wild type were included as controls. **(B)** Quantification of *uidA* and *nat* copy number in clones 33, 36, 40, 72, and 78. Gus5 and wildtype strains were included as controls.

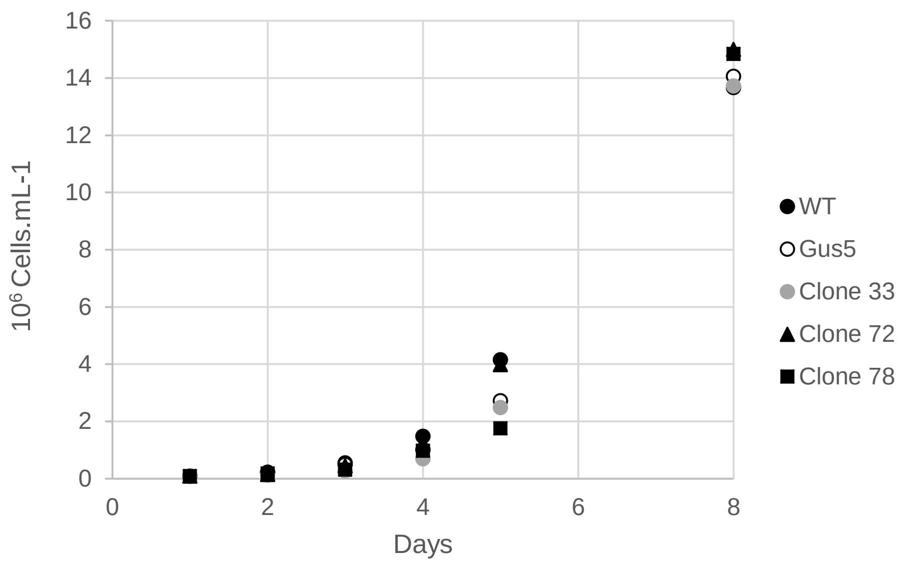

Supplementary Figure 11. Representative growth curves for various strains. WT was included as a control. The Y-axis indicates the cell density, expressed as millions per mL.

NAT-T2A-GUS	*uidA* copy	*NAT* copy	GUS Level
Clone 27	2.74	2.08	0
Clone 28	21.73	23.42	4
Clone 29	1.07	2.53	2
Clone 30	3.99	3.08	4
Clone 31	23.63	19.13	4
Clone 32	3.2	1.13	2
Clone 33	1.3	1.1	2
Clone 34	6.13	4.6	2
Clone 59	0.98	1.03	0
Clone 60	0.92	0.93	0
Clone 61	2.33	2.16	0
Clone 62	4.5	3.25	0
Clone 63	11.1	12.6	4
Clone 64	3.18	2.91	2
Clone 65	2.9	3.26	0
Clone 66	5.41	3.1	2
Clone 67	1.88	1.91	3
Clone 68	8.44	5.94	4
Clone 69	4.91	5.24	3
Clone 70	15.93	9.51	4
Clone 71	2.43	2.18	0
Clone 72	1.06	1.01	4
Clone 73	1.14	1.09	0
Clone 74	4.79	2.74	4

Supplementary Figure 12. Table of clones in NAT-T2A-GUS indicating the *uidA* and *NAT* copy number and GUS level.

GUS-T2A-NAT	*uidA* copy	*NAT* copy	GUS Level
Clone 21	2.25	3.67	1
Clone 22	3.87	3.43	1
Clone 23	30.0	32.67	4
Clone 24	2.86	2.87	1
Clone 25	8.8	11.1	4
Clone 26	3.77	3.78	1
Clone 35	1.87	2.97	0
Clone 36	0.91	0.92	0
Clone 37	2.1	2.26	0
Clone 38	4.67	4.9	2
Clone 39	16.14	16.32	2
Clone 40	0.93	0.93	0
Clone 41	2.5	2.44	0
Clone 42	3.29	3.19	0
Clone 43	2.49	3.48	1
Clone 44	6.34	5.18	0
Clone 45	2.92	2.83	0
Clone 46	22.88	21.06	2

Supplementary Figure 13. Table of clones in GUS-T2A-NAT indicating the *uidA* and *NAT* copy number and GUS level.

NAT-P2A-GUS	*uidA* copy	*NAT* copy	GUS Level
Clone 5	1.16	1.64	2
Clone 6	11.55	9.63	4
Clone 7	22.87	20.34	4
Clone 9	17.45	20.6	4
Clone 10	19.8	20.03	4
Clone 11	1.99	0.94	3
Clone 12	5.1	3.51	3
Clone 75	17.41	13	4
Clone 76	8.94	8.23	4
Clone 77	26.36	30.72	4
Clone 78	0.95	0.93	3
Clone 79	8.18	7.6	4
Clone 80	7.95	8.2	4
Clone 81	8.56	7.8	4
Clone 82	31.54	32.32	3
Clone 84	1.84	1.48	3
Clone 85	10.15	8.4	3
Clone 86	1.89	1.93	0
Clone 87	8.60	8.15	3
Clone 88	6.53	6.75	4
Clone 89	2.62	2.52	2
Clone 90	9.2	11.6	0
Clone 91	1.28	1.29	0
Clone 92	1.27	1.28	0
Clone 93	5.79	4.11	3
Clone 94	20.34	13.61	3
Clone 95	4.79	2.73	4
Clone 96	24.14	19.68	4
Clone 97	26.84	27.47	4
Clone 98	5.63	3.54	2
Clone 99	2.94	1.04	1

Supplementary Figure 14. Table of clones in NAT-P2A-GUS indicating *uidA* and *NAT* copy number and GUS level.

GUS-P2A-NAT	*uidA* copy	*NAT* copy	GUS Level
Clone 13	3.7	3.64	0
Clone 14	2.23	3.28	0
Clone 15	41.43	47.35	4
Clone 16	7.46	8.14	3
Clone 17	7.71	7.27	3
Clone 18	5.35	5.42	2
Clone 19	18.55	15.94	4
Clone 20	4.0	4.24	1
Clone 47	6.95	6.74	1
Clone 48	26.95	28.78	4
Clone 49	5.07	5.41	2
Clone 50	2.45	3.67	0
Clone 51	1.9	1.82	0
Clone 52	3.7	3.63	0
Clone 53	2.09	2.09	0
Clone 54	1.8	1.8	0
Clone 55	14.49	12.05	2
Clone 56	1.68	1.69	0
Clone 57	2.01	1.91	0
Clone 58	1.98	3.14	0

Supplementary Figure 15. Table of clones in GUS-P2A-NAT indicating *uidA* and *NAT* copy number and GUS level.

Use	Target	Forward Primer	Reverse Primer
Integrity of expression cassette	PCR on polycistronic NTG, NPG, GTN and GPN constructs	CAGAAGACAATTGCGGAGAATC	GGATCCAGCGGAGCTCCA
ddPCR/ RT-ddPCR	*TBP*	ACCGGAGTCAAGAGCACACAC	CGGAATGCGCGTATACCAGT
	RPS	CGAAGTCAACCAGGAAACCAA	GTGCAAGAGACCGGACATACC
	uiDA	GAAGGGCGAACAGTTCCTGA	GCGAGGTACGGTAGGAGTTG
	NAT	TGGAGGTCACCAACGTCAAC	CAGGGCATGCTCATGTAGAG
Taqman probes	*TBP* (HEX)	AAGTTTGCCT+A+CATTGTGGAGCGC	
	RPS (HEX)	AGCAAAT+G+GCGGATCTCTCCC	
	uidA (6-FAM)	ATGAAGATGCGGACTTACGTGGCA	
	NAT (6-FAM)	CCTCTGCGGCCTGGACACC	

Supplementary Figure 16. Primer sequence information. For the *TBP* and *RPS* TaqMan probes, the LNA (locked nucleic acid), indicated by (+), have been added to respect the supplier's guidelines.
